# Accelerated mineralization of textile wastewater under 222 nm irradiation from Kr/Cl_2_ excilamp: an environmentally friendly and energy efficient approach

**DOI:** 10.1038/s41598-024-63012-z

**Published:** 2024-05-31

**Authors:** Kiran Ahlawat, Ramavtar Jangra, Ram Prakash

**Affiliations:** Department of Physics, Indian Institute of Technology Jodhpur, Jodhpur, Rajasthan 342037 India

**Keywords:** 222 nm, Far UV-C, Reactive black 5, Textile wastewater, COD, RB5 degradation, AOP, Pollution remediation, Nanoparticles, Photocatalysis

## Abstract

The textile dyeing and manufacturing industry is the major producer of significant amounts of wastewater that contain persistent substances such as azo dyes that require adequate remediation measures. Far ultraviolet at 222 nm light may provide an advantage for contaminants degradation as compared to conventional UV sources (254 nm). In this paper, the degradation of reactive black 5 (RB5) in artificial wastewater has been performed using a 222 nm Kr/Cl_2_ excimer source under direct photolysis and an advanced oxidation process using TiO_2_/H_2_O_2_. The solution pH, catalyst concentration, 222 nm intensity, initial concentration of dye, and addition of H_2_O_2_ influence the degradation rate constant. The molar absorption coefficient, quantum yield of RB5 at 222 nm and the electrical energy per order (EEO) from different treatment methods have been reported. RB5 shows 1.26 times higher molar absorption at 222 nm than at 254 nm. The EEO for excimer-222/H_2_O_2_ ($\sim$ 13 kWh/m^3^) is five times lower than that of the excimer-222/TiO_2_ process, which makes the process energy efficient. The degradation of wastewater has been carried out at three distinct pH values (2, 6, and 10), and the pH level of 10 exhibited the highest degree of degradation. The degradation rate in the alkaline medium is 8.27 and 2.05 times higher than in the acidic or ambient medium. Since textile effluent is highly alkaline, this result is significant, as no neutralization of the wastewater is required, and direct treatment is possible. A possible degradation pathway has been established based on Fourier transform infrared spectroscopy (FTIR) and high resolution mass spectroscopy (HRMS) analysis. The phytotoxicity of the treated wastewater has also been evaluated for its suitability for reuse in agriculture. The study reveals that the excimer-222/H_2_O_2_ treated wastewater significantly enhanced the germination percentage of Raphanus sativus seed (97%) compared to dye wastewater-grown seeds (75%). This work offers crucial information for future studies on the direct and indirect photolysis of azo dyes, as well as insight into the process of RB5 degradation under Kr/Cl_2_ excimer radiation.

## Introduction

The treatment of wastewater polluted with dye is a significant global issue, necessitating the adoption of various strategies for eradicating organic contaminants from the wastewater^1^. Different techniques are employed to eliminate organic pollutants from wastewater, such as adsorption^2^, membrane filtration^3^, biological treatment^4^, and advanced oxidation process (AOP)^5,6^. The extensively employed textile wastewater treatment techniques are ultraviolet (UV) and UV-based advanced oxidation processes (UV-AOP)^7^. The conventional technique uses a 254 nm low-pressure UV (LPUV) source. Some organic dyes can be degraded by direct photolysis under LPUV, while others need a UV/AOP process, like reactive dyes/azo dyes^8^. AOP is a promising approach that generates highly reactive hydroxyl radicals (·OH), which are potent and non-selective oxidants. These radicals effectively speed up the removal of hazardous contaminants^9^. Among various AOPs, UV radiation in conjunction with hydrogen peroxide (H_2_O_2_), commonly referred to as UV/H_2_O_2_ AOP^10^, and the heterogeneous photocatalytic oxidation, particularly UV/TiO_2_, are drawing much attention^11^.

The use of TiO_2_ is a more attractive choice to utilize as photocatalysts in environmental clean-up operations because of its high chemical stability, high reactivity, low cost, non-toxic and insoluble nature^12^. The ultimate mineralization products of the photocatalytic oxidation process are carbon dioxide (CO_2_), water (H_2_O), and other mineral acids, making it an environmentally benign process^13^. However, it is removed from some home-use products as tooth paste due to concerns regarding biocompatibility^14^. The synthesis and extraction of the catalyst post-treatment is a time-consuming and costly operation that should be reduced to a minimum. The UV/H_2_O_2_ AOP can serve as a viable option for this purpose. UV rays can sanitize the water for point of use without additional chemicals and has the potential to eliminate organic contaminants. The choice of UV wavelength for H_2_O_2_ photolysis is crucial, as it influences the effectiveness of ·OH generation during the process, that further affect the oxidation of contaminants that are not decomposed with the direct photolysis (DP) method. In the conventional photolysis process, the photolysis of H_2_O_2_ is initiated by the absorption of a photon with a wavelength of 254 nm. Bar-Niv et al.^15^ conducted an experiment on the degradation of 700 ppm phenol using UV_254_/H_2_O_2_. They used a concentration of H_2_O_2_ as high as 300 ppm. The results showed that only 20% of the phenol was degraded after 20 h of treatment. Cedat et al.^16^ conducted a study on the degradation of 1.3 mg/L of estrogens using UV_254_/H_2_O_2_. They used 40 ppm of H_2_O_2_ and observed a 99% degradation with a UV radiation dose of 1000 mJ/cm^2^. As reported by Yin et al.^17^, the H_2_O_2_ has limited absorption at a wavelength of 254 nm, and photolyzed only 10% to produce ·OH. Following degradation, the elimination of leftover oxidant from the water matrix necessitates the use of a carbon filter, hence increasing the overall cost of the treatment.

One potential alternative is employing shorter wavelengths, specifically far UV-C (200–230 nm), which has been found to have enhanced efficiency in generating ·OH through the reaction with H_2_O_2_^18,19^. The Kr/Cl_2_ excilamp, which primarily emits radiation at a wavelength of 222 nm, has been identified as a promising new source of UV radiation for disinfection. The molar absorption of H_2_O_2_ at a wavelength of 222 nm is 97.7 M^−1^ cm^−1^, while at 254 nm, it is only 18.6 M^−1^ cm^−1^^19^. The utilization of this new light source with a specific wavelength has the potential to significantly contribute to the expedited breakdown of contaminants present in wastewater^20^. Kr/Cl_2_ excilamp irradiation have the ability to disinfect water at the point of use without the need for further chemicals, and they have the capability to remove organic contaminants. One more advantage of these shorter wavelength sources is that they are safer for human skin and eyes than conventional sources^21,22^. Using XeBr, KrCl, and Cl_2_ excilamps, Murcia et al.^23^ conducted dye photodegradation experiments and reported that KrCl excilamps exhibited the highest removal rate, followed by XeBr and Cl_2_ excilamps. The same research group compared two excilamps in a UV/H_2_O_2_ process in a different study, and the results showed that excimer technology could potentially be a great substitute for traditional techniques^24^.

Gen et al.^25^ have reported the degradation of trichloroacetic acid (TCAA) using Kr/Cl_2_ excilamp and reported 78% of TCAA decomposition within 200 min. The UV power density of their excilamp was 0.12 mW/cm^2^ at the input power of 60 W. The low UV power density and high input power need to be taken care to minimize the degradation time and improve the energy efficiency of the system. Li et al.^26^ examine the efficiency of Kr/Cl_2_ excilamp for the disinfection of waterborne pathogens. Kr/Cl_2_ excilamp developed by them provides an intensity of 0.03–0.3 mW/cm^2^ by utilizing the power from 15 to 60 W. Xu et al.^8^ reported the degradation of 46 organic micropollutants under direct photolysis by Kr/Cl_2_ excilamps and concluded that nitrate/nitrites present in the water matrix improves the degradation of non-nitrogenous, aniline and trizine OMP, while higher quantum yield occurs for nitrogenous OMPs. So, the degradation of different organic pollutant depends upon the molar absorption coefficients and the quantum yield at the wavelength used. Kr/Cl_2_ excilamps are extensively researched as an effective source for the degradation of organic contaminants but a narrow band source with self-cooling has not been discussed. Furthermore, the electrical energy per order has not been reported yet for the Kr/Cl_2_ excilamps to compare the cost effectiveness of the overall degradation process with traditional processes. The RB5 organic azo dyes’ molar absorption coefficients and quantum yield at 222 nm have not yet been investigated, and the excimer-222/H_2_O_2_ process breakdown mechanism and byproducts need to be understood. It is further necessary to investigate the toxicity of textile wastewater after treatment in order to determine whether it may be used again in agriculture.

In this paper, we have reported an energy efficient DBD-based 222 nm Kr/Cl_2_ excilamp emitting narrow spectral band with high UV power density. The developed Kr/Cl_2_ excilamp has been employed for the degradation and mineralization of artificial wastewater. Accordingly, the developed source has been utilized for the degradation of RB5 azo dye in artificial wastewater by DP, excimer-222/H_2_O_2,_ and excimer-222/TiO_2_ and the obtained results are compared with the LPUV/AOP. The effects of different concentrations of catalysts loading, initial concentrations of dye, solution pH, and different concentrations of H_2_O_2_ are examined at different UV fluencies for the degradation of artificial wastewater. The molar absorption coefficient, quantum yield and EEO are reported at 222 nm and 254 nm for RB5 dye. The removal of dye toxicity from samples is also carried out using the chemical oxygen demand (COD) test, and the results are correlated. The excimer 222 nm and H_2_O_2_ based degradation mechanism and a possible degradation pathway have been proposed. To determine the toxicity of dye wastewater treated using various techniques, Raphanus sativus seeds are germinated in soil using the treated artificial wastewater and deionized water.

The following are the key elements of uniqueness in the present research:

1. The molar absorption coefficient and quantum yield of azo dye has been reported for the first time for 222 nm wavelength.
2. A Kr/Cl_2_ excilamp has been developed and optimized which emits 222 nm UV-C light and will be safer for human exposure. A narrow and intense spectral band has been obtained that can be utilized for the photolysis of H_2_O_2_ for the enhanced production of hydroxyl radicals.
3. By adding H_2_O_2_ as a radical promotor (less than 10 ppm) in excimer-222/AOP, accelerated degradation of organic dye is reported as compared to the photocatalyst loading. This process has eliminated the recovery step of catalysts from the treated wastewater.
4. Instead of utilizing DI water as a solvent, RB5 dye has been dissolved in sea water matrix for the practical applicability of the proposed method. A degradation mechanism under excimer-222/H_2_O_2_ has been discussed to adequately understand the degradation reaction kinetics.
5. One of the primary distinctive features of this study is to utilize the post-treated wastewater for phytotoxicity analysis on seed germination to assess its prospective use in agriculture.

## Experimental setup

### Materials and far UV-C 222 nm source

The information about the different materials used in this study is included in Sect. 1.1 of the supplementary information (SI). The information on the development process of the Kr/Cl_2_ excilamp is explained in our previous work^27^ and also provided in Sect. 1.2 of SI.

**Figure 1:**
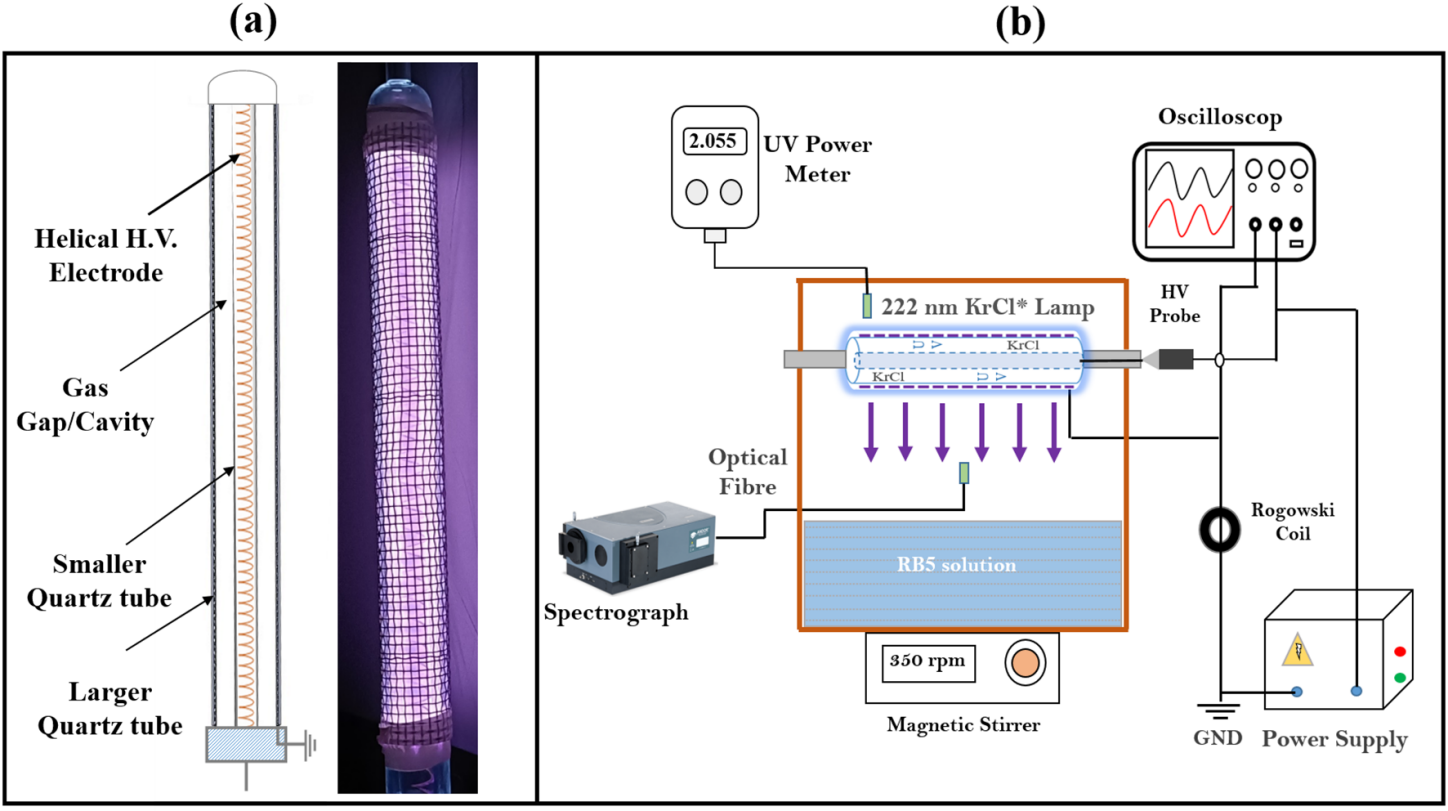
(**a**): 2-D schematic and original view of the KrCl* (222 nm) excimer light source. (**b**) Experimental setup of developed DBD Kr/Cl_2_ excilamp with all electrical and optical measuring equipment. The optical emission spectra and UV 222 intensity are recorded from the centre of the Kr/Cl_2_ excilamp.

Figure 1a shows the 2-D schematic and original view of the developed Kr/Cl_2_ (222 nm) excilamp. When subjected to high voltage, the excimer source triggers gas excitation, producing KrCl* excimer molecules. In an instant of nanoseconds after excitation, the excimer molecules undergo decomposition and subsequently emit ultraviolet (UV) photons with a specific wavelength of 222 nm, accompanied by weaker transitions occurring at various wavelengths. The developed excilamp had a length of 15 cm and an outside diameter of 1.6 cm. This measurement provides a surface area of approximately 75 cm^2^, adequate for emitting UV radiation. The optical parameters of the exciplex source were analysed using an Andor PMT-based monochromator (SR-500i-B1) equipped with an optical fibre (200 µm, ocean optics). The optical spectra of the far UV-C exciplex source from 200 to 300 nm were recorded using a grating with 2400 grooves/mm. For the measurement of the incident irradiation ${E}_{0}$ (in mW/cm^2^) from the excimer source in air, a Hamamatsu make UV power meter (C9536) has been used with a sensor head (H9535-222) pre-calibrated at the 222 nm wavelength. The average UV fluence rate (${E}_{avg}$) delivered to the wastewater samples has been calculated by including all the factors that influence the intensity i.e., water factor (WF), petri factor (PF), divergence factor (DF), and reflection factor (RF) as shown in the equation,

1 $$E_{avg} = E_{0} \times WF\left( \lambda \right) \times PF\left( \lambda \right) \times DF \times RF$$

All these parameters are calculated using the standard method given by Bolton and Linden^28^, and reported as WF is 0.985, PF is 0.973, RF is 0.90. A complete experimental setup, for the optical and electrical characterization of the developed Kr/Cl_2_ excimer source is shown in Fig. 1b.

### Preparation and characterization of TiO_2_

The influence of phase and crystalline size on the photocatalytic activity of a catalyst is significant^29,30^. The TiO_2_ nanoparticles were synthesised using the sol–gel technique, and a detailed procedure for their preparation is explained in our previous studies^31,32^. To confirm the formation of nanoparticles, the TiO_2_ suspension was subjected to further annealing at a temperature of 450 °C for 3 h. The resulting powder was then examined using X-ray diffraction (XRD) and X-ray photon spectroscopy (XPS).

The Tauc equation and Kubelka–Munk technique were employed to determine the optical bandgap (${E}_{g}$) energy of the synthesised nanoparticles^33^. XRD (D-8 Advance, Bruker) equipped with Cu-Kα radiation (λ = 1.54 Å) was performed to confirm the phase of the TiO_2_ nanoparticle, data were taken between 2 theta values of 20° and 80° with a step size of 0.02°. The field emission scanning electron micrograph (FE-SEM, Apreo 2, Thermo Fisher Scientific, USA) and high-resolution tunnelling electron microscope (HRTEM, Talos F200X G2, Thermo Fisher Scientific, USA) confirms the crystalline size of the synthesized nanoparticles. The FE-SEM characterization has been performed with 10 kV high voltage in secondary electrode (SE) mode. X-ray photoelectron spectroscopy (XPS: Monochromatic Al-Kα source, Thermo Fisher Scientific, USA) studies were performed to observe the surface property and chemical states of the synthesized material. For the XPS characterization, monochromatic Al-Kα source (*hv* = 1486.6 eV) with a hemispherical analyser and a 128 channel plate detector have been used.

### Procedure and analysis of pollutant degradation

The DBD Kr/Cl_2_ excilamp was employed to study the degradation and mineralization of RB5 dye in artificial wastewater under different conditions. To validate the developed reactor for industrial applications, artificial wastewater was used for the experimentation. For the same, diluted sea water (pH = 10, conductivity $\sim$ 7370 µS/cm, and TDS $\sim$ 3850 ppm) is treated with excimer 222 and LPUV with TiO_2_ and H_2_O_2_ loading. Total dissolved solids (TDS) refers to the quantitative measurement of all inorganic and organic components that are present in a liquid in molecular, ionized, or micro-granular suspended form. Generally, the electrical conductivity of textile wastewater was found to be in the range of 4400–8700 µS/cm. Diluted sea water was considered as a surrogate of textile wastewater because of the presence of various salts. Furthermore, textile industries uses various salts for the wet dying process to enhance the penetration of dye molecules into the fabric as well as for the enhanced dying efficiency^34^. In the present study, diluted seawater along with RB5 dye is considered as a textile wastewater.

A solution containing different concentrations of RB5 dye (10 mg/L, 50 mg/L, and 90 mg/L) in artificial wastewater was chosen as a model pollutant. RB5 is chosen for this study because of its complex chemical structure as well as its molar absorption coefficients and quantum yield is unknown for 222 nm. An acrylic chamber of one cubic feet was used to integrate the Kr/Cl_2_ excilamp for dye degradation experimentation. 100 ml of artificial wastewater solution was treated for each test and 2 ml samples from suspension were withdrawn at regular time intervals and were immediately centrifuged at 3000 rpm for 15 min to completely remove catalyst particles if they were loaded. A calibration curve for known RB5 concentrations is plotted using the standard solution (see Figure S1) to measure the amount of RB5 after different processes.

To optimise the loading of the catalyst, varying concentrations of TiO_2_ catalyst (0.5 g/L, 0.75 g/L, 1 g/L, 1.25 g/L, and 1.5 g/L) were introduced. The resulting suspension was then magnetically stirred for 30 min in a dark environment. This was done to ensure the thorough equilibration of the adsorption and desorption of RB5 on the surface of TiO_2_. In most cases, studies were conducted at ambient pH of the solution during the photocatalytic reaction. To alter the initial pH for acidic or alkaline studies, the appropriate amount of H_2_SO_4_ or NaOH was added accordingly. The experiments were conducted under standard temperature and pressure conditions. The transfer of radiative heat from the excilamp to the treated suspension can be computed using fluid modelling studies^35^. Each experiment was replicated multiple times to ensure preciseness, and the median values were plotted, wherever possible. Kruskal–Wallis test was performed to compare quantitative variables which were not normally distributed.

The concentration of RB5 dye at its maximum absorbance (${\lambda }_{max}$ = 598 nm) was measured using a UV–Vis spectrophotometer (Cary 4000, Varian). High-resolution mass spectrometry (HR-MS) and Fourier transform infrared spectroscopy (FTIR) were employed to analyse the potential degradation mechanism. The COD test has been used to investigate dye mineralization by measuring the oxygen demand for the breakdown of organic pollutants. COD is used in wastewater treatment plants to quantify the amount of oxygen needed to decompose organic matter (pollutants).

The molar absorption coefficient (ε) and quantum yield (Ø_λ_) for RB5 dye are determined using the following relationships^36^,

2 $$\varepsilon \left( {{\text{M}}^{ - 1} {\text{cm}}^{ - 1} } \right) = \frac{A}{lC}$$

3 $$\O_{\lambda } = \frac{k\left( \lambda \right)}{{E_{avg} \left( \lambda \right)}} \frac{1}{{2.303 \varepsilon_{\lambda } }}$$

where A is the absorbance of the sample at a particular path length, C is the concentration of target compound (M), *l* is the path length (cm), k(λ) is the pseudo first order rate coefficient (s^−1^), ${E}_{avg}$ (λ) is the average incident photon irradiance. The pseudo-first-order kinetic model for RB5 degradation is shown in Eq. (7). The dye degradation efficiency (%), EEO, and COD reduction are measured using the following expressions,

4 $$Degradation\;efficiency \left( \% \right) = \frac{{C_{0} - C_{t} }}{{C_{0} }} \times 100$$

5 $$EEO\left( {\frac{kWh}{{m^{3} }}} \right) = \frac{P \times t \times 1000}{{V \times 60 \times \log \left( {\frac{{C_{0} }}{{C_{t} }}} \right)}}$$

6 $$COD\;reduction \left( \% \right) = \frac{{COD_{0} - COD_{t} }}{{COD_{0} }}$$

7 $$ln \left( {\frac{{C_{0} }}{{C_{t} }}} \right) = k \times t$$

where ${C}_{0}$ and ${C}_{t}$ are the initial and final concentrations of the RB5 dye (in mg/L), P is the consumed power of Kr/Cl_2_ excilamp (in kW), V is the treated suspension volume (L), t is the treatment/exposure time (in hour), ${COD}_{0}$ and ${COD}_{t}$ are the initial and final value of chemical oxygen demand (in mg/L), k is the first order rate coefficient.

### Evaluation of the reusability and toxicity of the dye wastewater

The reusability of treated wastewater was evaluated by germination of Raphanus sativus seeds to test the toxicity of untreated and treated artificial organic wastewater. The process of seed germination was investigated utilising various water samples, including deionized water, artificial wastewater, LPUV DP, excimer-222 DP, excimer-222/TiO_2_ (1 g/L), and excimer-222/H_2_O_2_ (10 ppm) treated artificial wastewater. Prior to employing the treated solutions for germination, their pH was neutralized. The Raphanus sativus seeds were planted directly in the soil, and untreated and treated wastewater were used to irrigate the soil. The purpose was to observe the impact of various water samples on the growth of the shoot and root of the seeds. This study was conducted over a period of 3 d to investigate the effects of treated and untreated organic wastewater on the germination rate, root length, and shoot length of Raphanus sativus seeds. Germination percentage has been calculated by using the following relationship,

8 $$Germination\;percentage = \frac{Number\;of\;seeds\;germinated}{{Number\;of\;seeds\;sown}} \times 100$$

## Results and discussion

### Excilamp characterization

**Figure 2:**
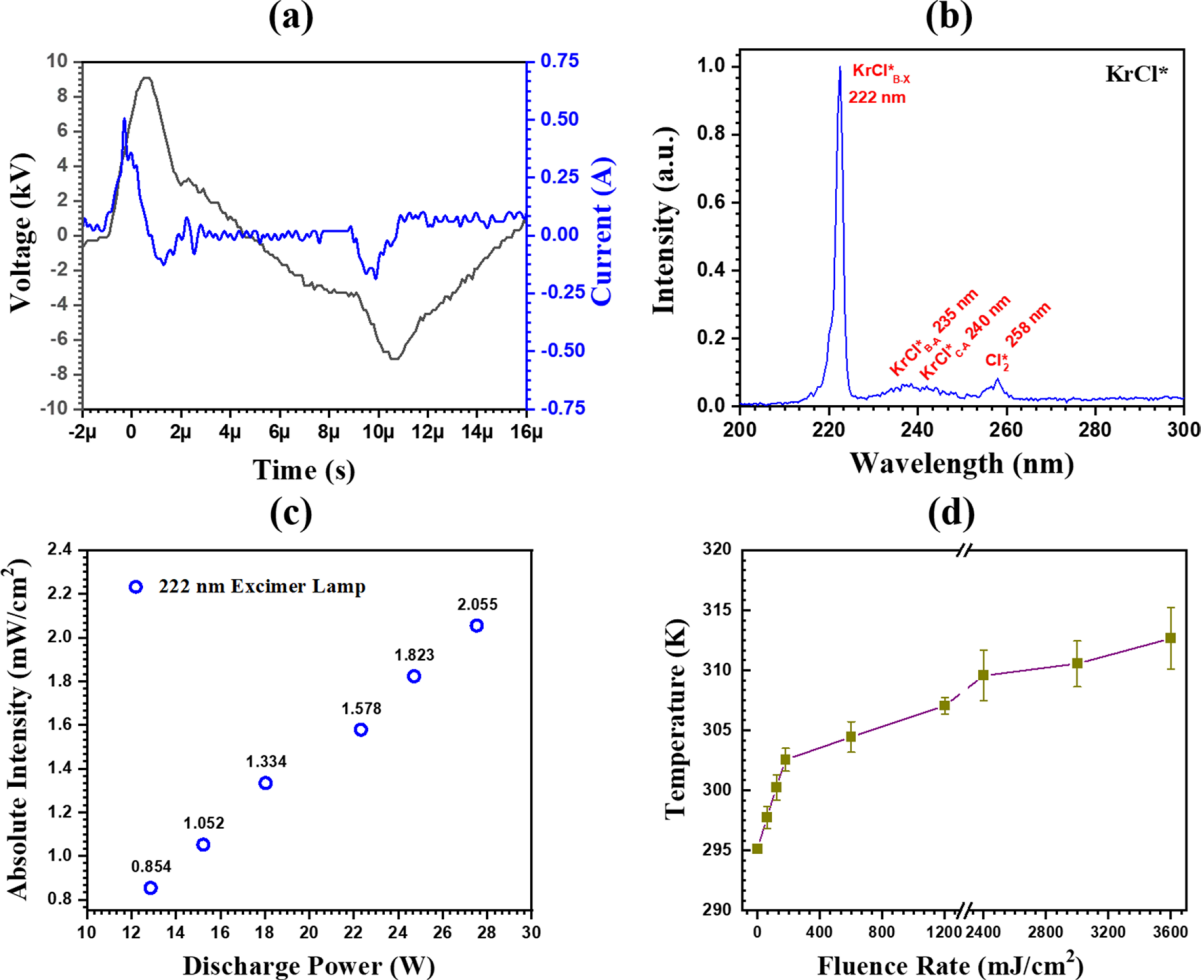
(**a**) Typical voltage and current waveforms of the DBD Kr/Cl_2_ excilamp, (**b**) OES of Kr/Cl_2_ excilamp, (**c**) The absolute UV intensity of the Kr/Cl_2_ excilamp with respect to discharge power, and (**d**) The relationship between the temperature change of the treated suspension and the excimer 222 nm fluence rate.

Figure 2a shows the typical voltage and current waveforms of the DBD Kr/Cl_2_ excilamp at an applied parameters of 9 kV/25 kHz. The DBD Kr/Cl_2_ excilamp discharge power is calculated by multiplying the measured voltage and current values, followed by their integration over a given time period^37^. At optimized parameters, the discharge power of the DBD Kr/Cl_2_ excilamp is found to be 15.23 W with 1050 µW/cm^2^ of UV output. A further increase in input power linearly increases the UV output of the DBD Kr/Cl_2_ excilamp. Figure 2b shows the optical emission spectra (OES) of the DBD Kr/Cl_2_ excilamp. A prominent spectral band at 222 nm has been observed, which corresponds to the B-X transition of KrCl* excimer. Additionally, there are weaker transition bands observed at 235 nm (B-A transition of KrCl* excimer), 240 nm (C-A transition of KrCl* excimer), and 258 nm (${Cl}_{2}^{*}$ transition). In addition to the 222 nm spectral band, these weak bands contribute to the breakdown of contaminants by promoting the production of active radicals.

The UV power density (absolute intensity at 222 nm) of the developed DBD Kr/Cl_2_ excilamp as a function of discharge power (W) is shown in Fig. 2c. It is observed that the intensity exhibits a linear relationship with the increase in discharge power (R^2^ > 0.99), and it takes around 30 s to stabilise the UV intensity. Until specifically mentioned, the studies addressing the degradation of RB5 dye were conducted with an absolute UV intensity of 1050 mW/cm^2^, which was optimised based on gas pressure, applied voltage, and excilamp heating. The temporal variation of temperature of the treated dye suspension in relation to the far UV-C fluence rate is depicted in Fig. 2d. Even after 3600 mJ/cm^2^ (or 60 min) of excimer 222 nm exposure, the temperature of the dye solution remains well below 315 K, indicating the non-thermal nature of excilamp and the treated suspension.

### Characterization of the material

**Figure 3:**
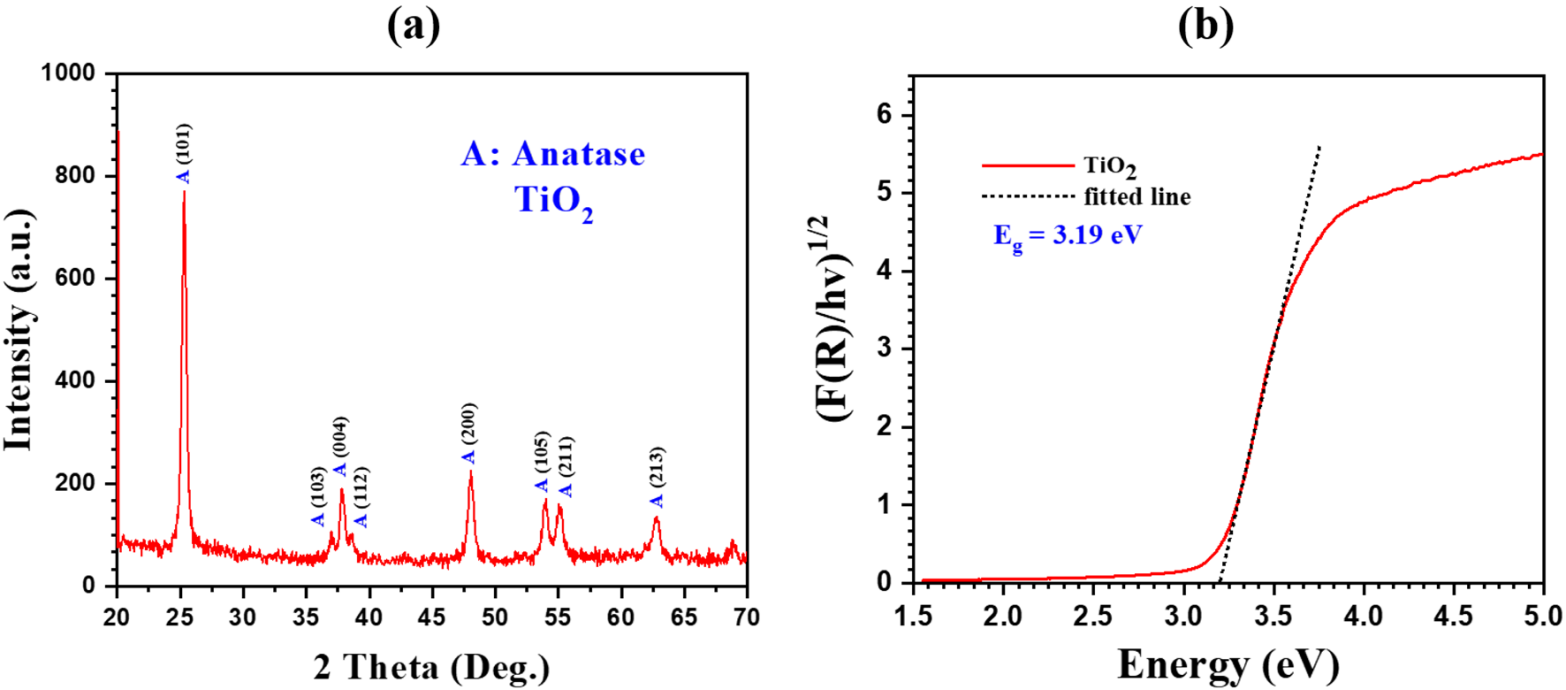
(**a**) XRD pattern and (**b**) Kubelka-Munk plot of the synthesized TiO_2_ nanoparticles.

The XRD pattern of TiO_2_ nanoparticles is shown in Fig. 3a. The most intense characteristic peaks in the XRD pattern at 25.21° which corresponds to the (101) crystal plane of TiO_2_. The minor peaks appeared at 36.97°, 37.78°, 38.52°, 48.01°, 53.94°, 55.01°, and 62.64°, which correspond to (103), (004), (112), (200), (105), (211), and (213) crystal planes of TiO_2_, respectively^38^. The XRD spectra of synthesized material reveal the pure anatase phase of TiO_2_ nanoparticles. Further, the diffuse reflectance spectra were recorded to investigate the optical bandgap of the TiO_2_. The Kubelka-Munk function is represent as $F\left(R\right)= \frac{{(1-R)}^{2}}{2R},$ where F(R) and R represent as comparable to the absorption coefficient ($\alpha )$ and mode of the reflectance and ${(F\left(R\right)h\nu )}^\frac{1}{2}$ vs $h\nu$ plotted and shown in Fig. 3b. The power ½ reperesents the indirect band gap nature for the synthesised materials. To get the band gap, the linear extraplottation of the ${(F\left(R\right)h\nu )}^\frac{1}{2}$ = 0 drawn on the x-axis. The obtained band gap for TiO_2_ is 3.19 eV which corresponds to the anatase phage^39^. According to the literature, this phase of TiO_2_ nanoparticles is the most suitable for photocatalytic applications in degrading dyes^40^.

**Figure 4:**
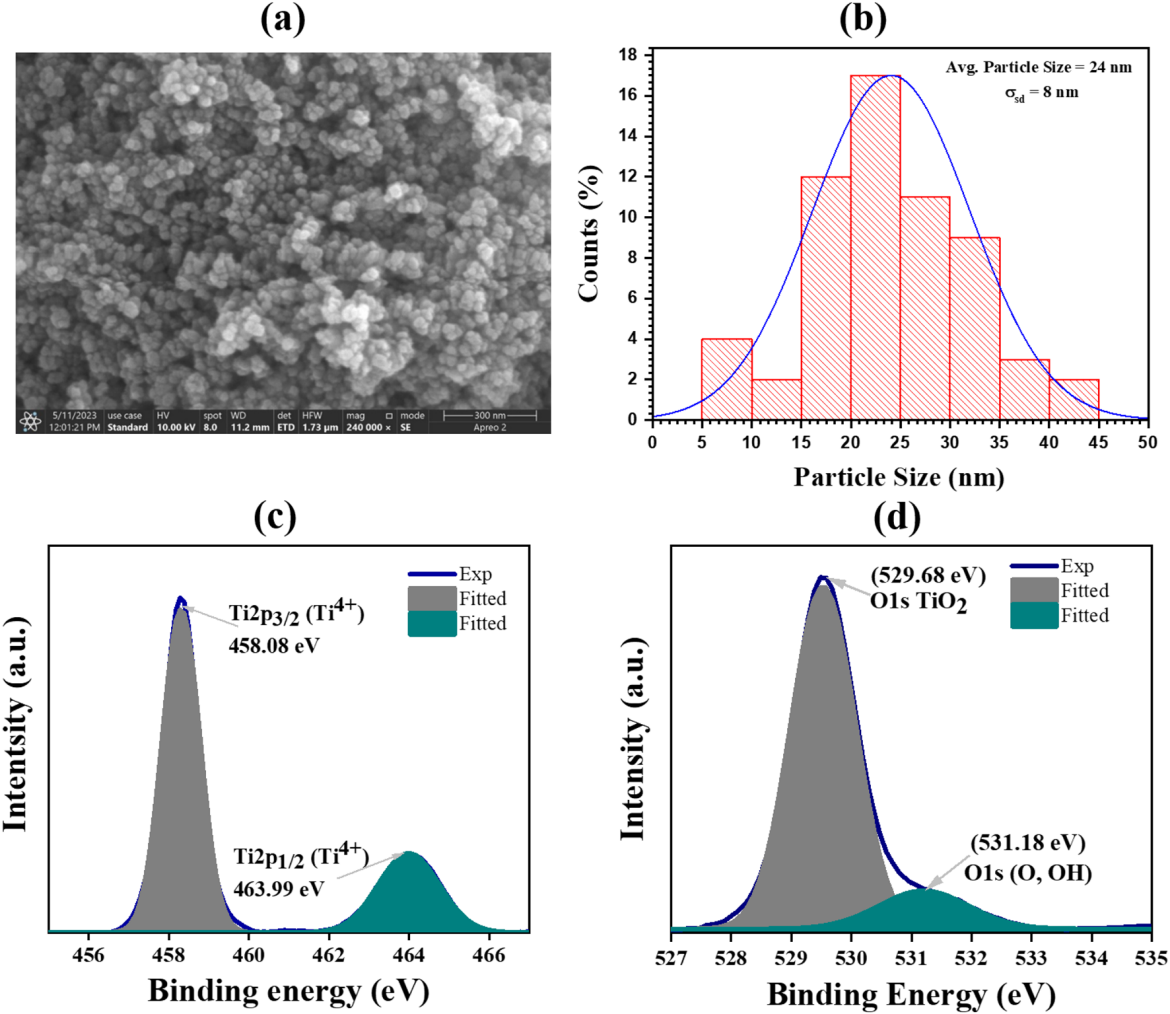
FE-SEM image of the synthesized (**a**) TiO_2_ nanoparticles, (**b**) histogram distribution of TiO_2_ nanoparticles for calculating average particle size. High-resolution XPS spectrum of (**c**) Ti 2p and (**d**) O1s in synthesized TiO_2_.

To evaluate the size and morphological characteristics of the synthesized nanoparticles, the TiO_2_ nano-powder was subjected to FE-SEM analysis. The resulting image is depicted in Fig. 4a. The particle size of TiO_2_ was analysed using the image view software, with measurements conducted for more than 60 particles. Figure 4b display the historiographical depiction of this measurement. The Gaussian distribution was employed for size analysis. The nanoparticles have an average size of $\sim$ 24 ± 8 nm, as seen in Fig. 4b, making them appropriate for photocatalytic applications^41^. These distribution and XRD measurements confirmed that the synthesised TiO_2_ material are in the nanometer regime. The size of synthesized nanoparticles were also confirmed by the HRTEM analysis, and the average size was found between 25 and 35 nm (as can be seen from Figure S2).

The surface property and different chemical states of TiO_2_ nanoparticles have been evaluated using XPS. Figure 4c and d shows the XPS spectrum recorded for the synthesised TiO_2_. Two spectrums are observed: one for O 1s and the other for Ti 2p. The XPS spectrum of Ti 2p is depicted in Fig. 4c, displaying two distinct doublet peaks: Ti 2p3/2 and Ti 2p1/2, with binding energies of 458.08 eV and 463.99 eV, respectively. The presence of this doublet can be attributed to the spin–orbit coupling interaction^42^. The primary peak observed at 529.68 eV in Fig. 4d is attributed to the presence of TiO_2_. A broad peak at 531.18 eV may indicate that the adsorbed hydroxyl and oxygen ions are weakly bonded. In addition to Ti and O, no additional peaks are observed, indicating the high degree of phase purity of the synthesised TiO_2_ nanoparticles.

### Characteristics of RB5 dye under different wavelengths

**Figure 5:**
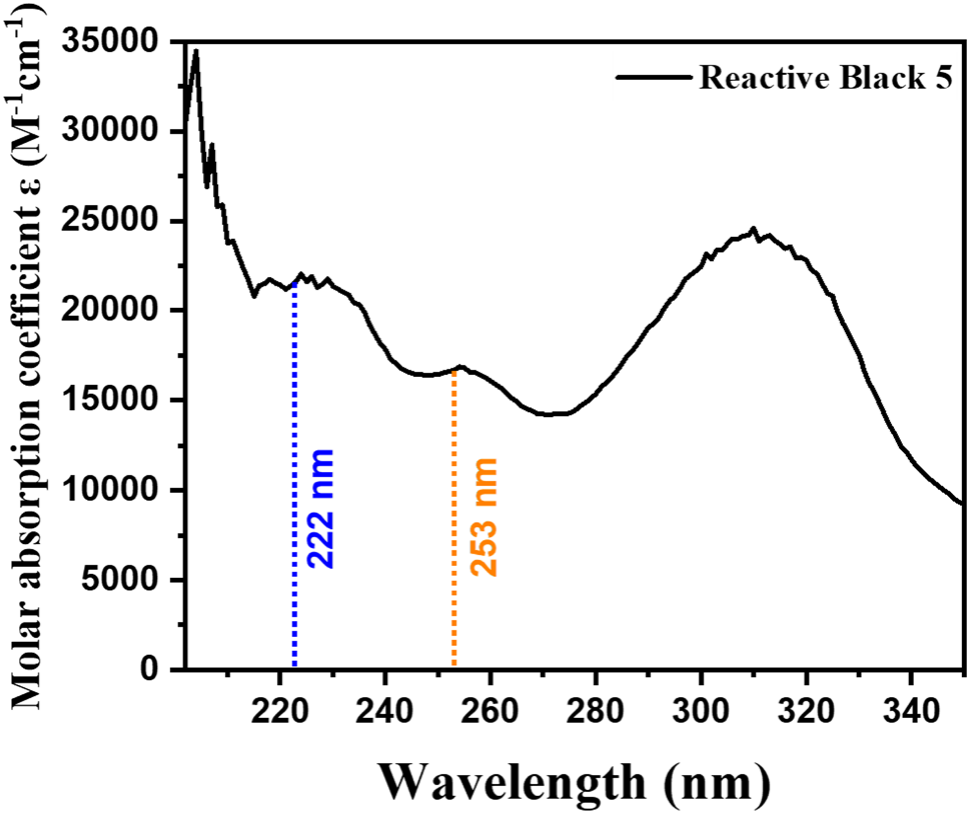
Molar absorption spectrum of RB5 dye.

Figure 5 shows the molar absorption spectra of the 50 mg/L RB5 dye suspension. It can be clearly seen from Fig. 5 that the molar absorption coefficient of RB5 dye at 222 nm (21,382 M^−1^ cm^−1^) is greater than 254 nm (16,870 M^−1^ cm^−1^), which means the RB5 dye will degrade 1.26 fold faster under 222 nm than 254 nm in case of direct photolysis. Also, the quantum yield at 222 nm is found be 0.0502, whereas the quantum yield at 254 nm is found to be 0.0018, which holds good promise for faster degradation under excimer 222/AOP. The molar absorption coefficient and quantum yield of RB5 at 222 nm and 254 nm wavelengths can be seen from Table S2.

A blank adsorption experiment was conducted in the dark, wherein a concentration of either 1 g/L of TiO_2_ or 10 ppm H_2_O_2_ was added to a dye suspension with a concentration of 50 mg/L. The results indicated minimal degradation (less than 2%) in the initial concentration of RB5. A comparable experiment was conducted under visible light conditions, revealing a decrease of 4% in the starting dye concentration after 60 min. This suggests that the utilized dye exhibits photostability within the visible spectrum, aligning with the earlier findings^43^.

### Parameters optimization

#### Effect of catalyst loading

**Figure 6:**
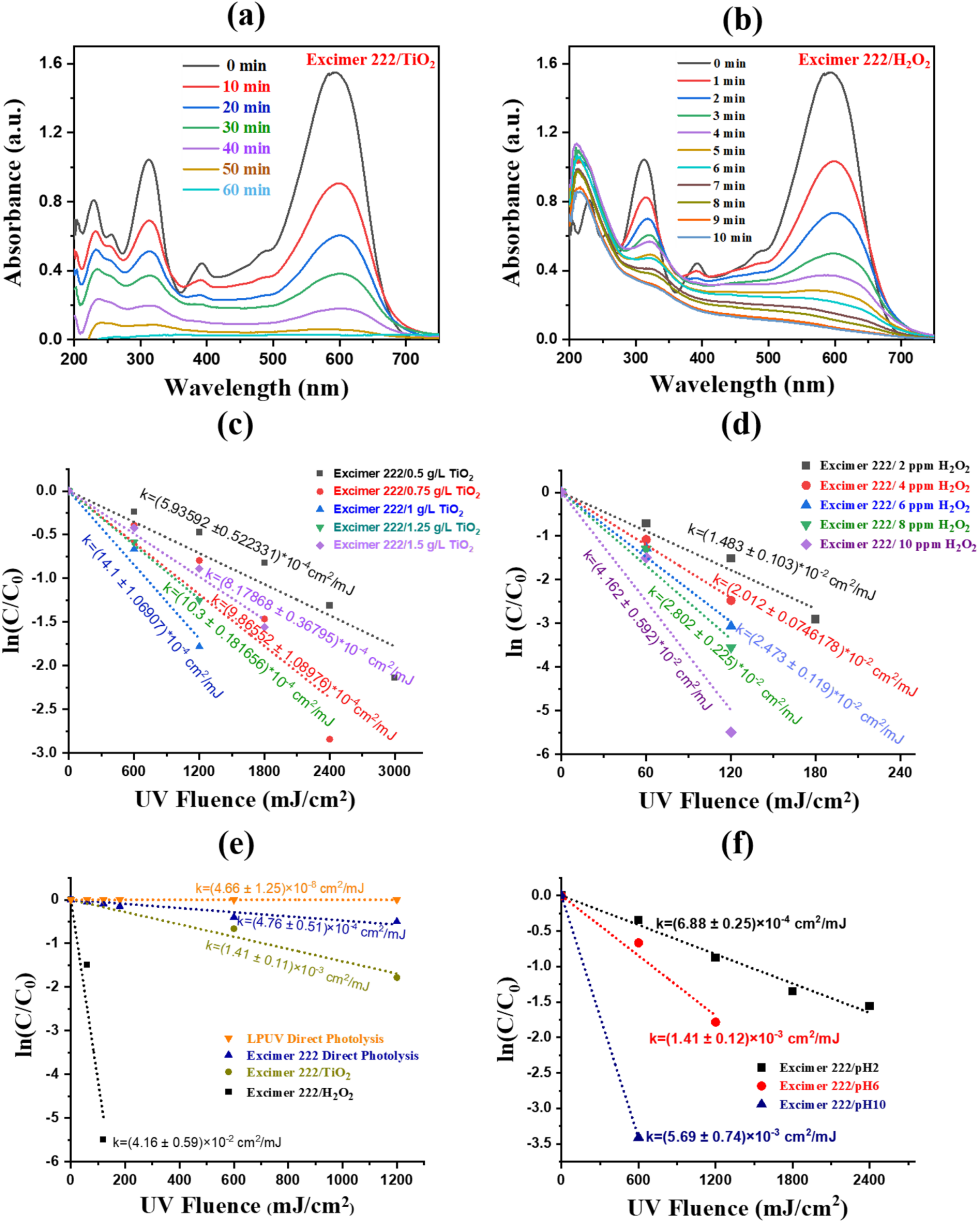
UV–Vis profile of RB5 dye solution before and after treatment of excimer 222 (**a**) with TiO_2_ (*C*_0_ = 50 mg/L, 1 g/L TiO_2_ and pH 10) and (**b**) with H_2_O_2_ (*C*_0_ = 50 mg/L, 10 ppm H_2_O_2_ and pH 10), in artificial wastewater. Effect of different loading concentrations of (**c**) TiO_2_ and (**d**) H_2_O_2_ w.r.t. the UV fluence of the excimer 222 nm on the degradation of RB5 dye at pH 10, (**e**) Comparison of RB5 degradation between excimer-222/TiO_2_, excimer-222/H_2_O_2_, and direct photolysis under DBD Kr/Cl_2_ excimer source (*C*_0_ = 50 mg/L, 1 g/L TiO_2_, and 10 ppm H_2_O_2_ and pH 10), (**f**) Effect of different pH values on RB5 dye degradation using DBD Kr/Cl_2_ excimer source (*C*_0_ = 50 mg/L, 1 g/L TiO_2_, and 10 ppm H_2_O_2_).

An important parameter for the photocatalytic degradation of any pollutant is the amount of the catalyst loaded into the suspension. Experiments were conducted at ambient pH by changing the catalyst amount from 0.5 to 1.5 g/L in an initial dye concentration of 50 mg/L to determine the most effective catalyst dosage. The UV–Vis profile of RB5 dye solution before and after treatment of excimer 222/TiO_2_ can be seen from Fig. 6a. The RB5 dye exhibits two prominent and broad absorption bands at 312 nm in the ultraviolet (UV) region and 598 nm in the visible region. The intensity of these bands decreases during exposure to the DBD Kr/Cl_2_ excilamp. The observed band at 598 nm indicates the presence of the azo double bond in the chromophore of RB5 dye. This band intensity experiences a quick drop upon exposure to excimer light with a wavelength of 222 nm in the presence of H_2_O_2_ and TiO_2_. The effect of catalyst loading on the degradation of dye is shown in Fig. 6c. It has been observed that the degradation rate constant (k) increases continuously from 5.93 × 10^–4^ to 1.41 × 10^–3^ cm^2^/mJ, with an increase in the catalyst loading from 0.5 to 1 g/L. By doubling the catalyst dose (from 0.5 to 1 g/L), the degradation rate constant becomes threefold. As the TiO_2_ loading increases, the absorption of UV-C photons also increases, leading to a higher production of ·OH and an increase in the absorbance of dye molecules^43^.

From this study, the optimal concentration of TiO_2_ catalysts for the degradation of 50 mg/L dye has been determined to be 1 g/L under DBD Kr/Cl_2_ excilamp with a degradation rate constant of 1.41 × 10^–3^ cm^2^/mJ. The reusability and stability of the synthesized catalyst have also been tested for up to six cycles (see Figure S3). The results reveal that the synthesized catalyst can be used for a number of cycles, demonstrating its stability and reusability in the practical wastewater applications.

**Figure 7:**
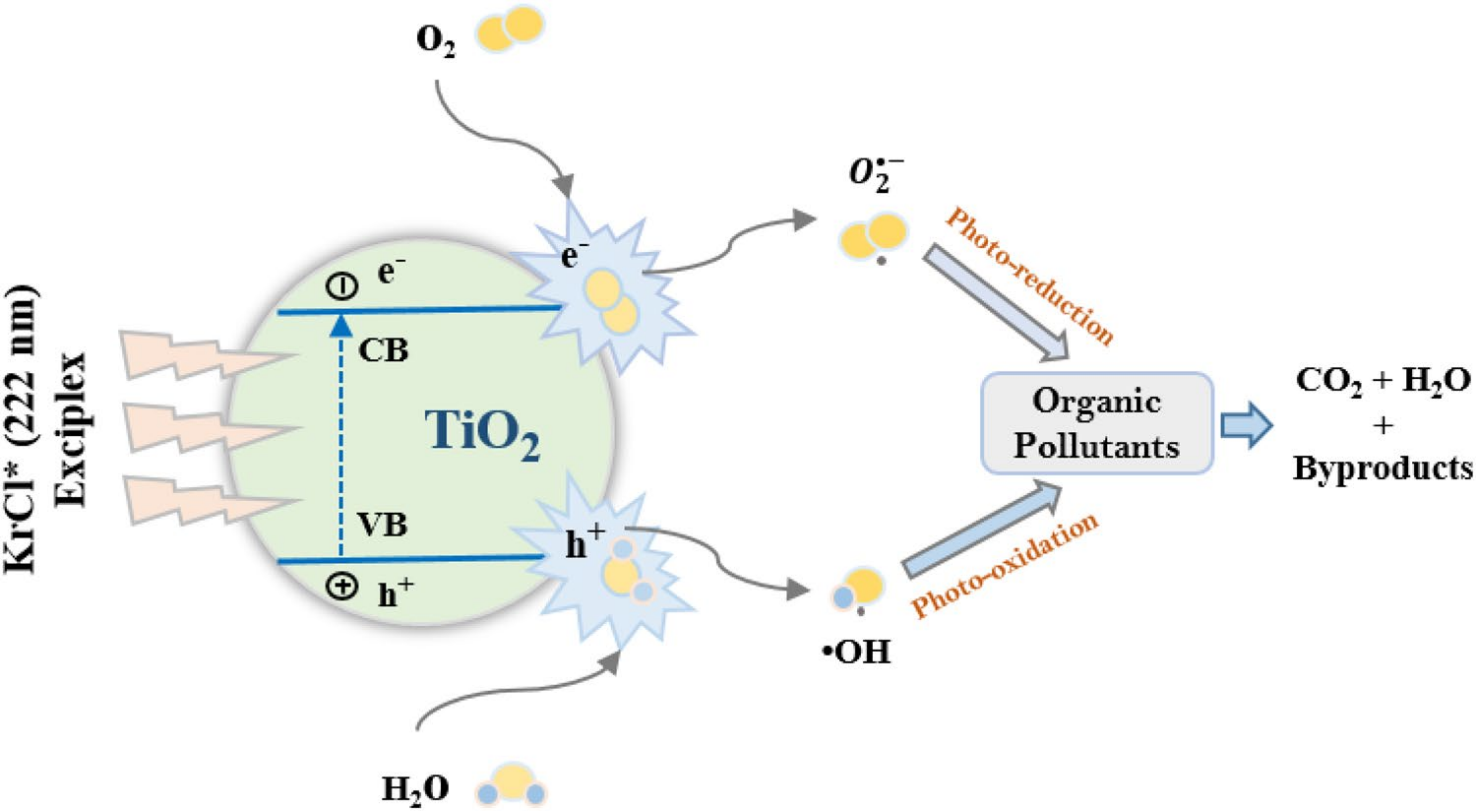
Photocatalytic mechanism action of TiO_2_ in organic pollutants treatment under the irradiation of Kr/Cl_2_ excilamp.

Figure 7 shows the photocatalytic mechanism action of TiO_2_ in organic pollutants treatment under the irradiation of Kr/Cl_2_ excilamp. The electrons and holes generated by photochemical processes undergo reactions with the oxygen and water molecules in their vicinity, forming superoxide radicals ($O_{2}^{ \cdot - }$) and ·OH. After a series of reactions, the $O_{2}^{ \cdot - }$ will subsequently generate ·OH. The organic substance will decompose into carbon dioxide, water, and various other by-products due to the action of ·OH. A detailed reaction mechanism of TiO_2_ has been provided in the SI in Sect. 1.3.

#### Effect of addition of H_2_O_2_

Figure 6b shows the UV–Vis profile of RB5 dye solution before and after treatment of excimer 222/H_2_O_2_. A weak band at 312 nm (in the UV-B region) has also been observed after decolourization, which is probably due to the presence of aromatic by-products in the reaction medium^43^. For 2 ppm concentration of H_2_O_2_, the degradation rate constant obtained is 1.48 × 10^–2^ cm^2^/mJ, whereas with further increase in the H_2_O_2_ concentration i.e. up to 8 ppm, the degradation rate does not show significant variations. Finally, the concentration of H_2_O_2_ increases to 10 ppm which is close to the limit of municipal UV/H_2_O_2_ systems^44,45^, a significant improve in the rate constant (four times faster than 2 ppm) was observed. Figure 6d shows the effect of different loading concentrations of the H_2_O_2_ over the degradation profiles of RB5. The degradation rate constant for RB5 in the case of excimer-222/H_2_O_2_ is 4.16 × 10^–2^ cm^2^/mJ, which is 29.5 times faster than the excimer-222/TiO_2_. From Fig. 6e, if we compare the DP of RB5 under LPUV and 222 nm, then 222 nm is more effective than LPUV due to its ability to produce more ·OH in water from the nitrates^19^. The degradation rate constant obtained in DP of RB5 under excimer 222 nm is 4.76 × 10^–4^ cm^2^/mJ. Upon comparison of the excimer-222/TiO_2_ and excimer-222/H_2_O_2_ with DP (222 nm) of the RB5 dye, it is found that the degradation rate of RB5 is 52 times faster in excimer-222/H_2_O_2_ case, whereas in the excimer-222/TiO_2_ the degradation rate of RB5 decreased and it is only 2.9 times faster than DP.

#### Effect of suspension pH

The pH of the treated suspension is a critical factor that significantly impacts the degradation of contaminants^46^. Three different pH values i.e. pH 2, pH 6, and pH 10 (solution) have been selected to see their impact on the degradation at a fixed TiO_2_ dosages of 1 g/L. The pH values are changed from alkaline (pH 10) to acidic (pH 2) by adding H_2_SO_4_ in the suspension. The impact of pH on the degradation of RB5 dye under acidic, ambient, and alkaline environments can be seen in Fig. 6f. The figure demonstrates that degradation occurs 8.27 times quicker in an alkaline medium compared to an acidic medium. The degradation rate constants of RB5 at pH values of 10 and 6 are 6.88 10^–4^ cm^2^/mJ and 1.41 10^–3^ cm^2^/mJ, respectively. These findings indicate that the degradation process occurs at a rate that is twice as fast in an alkaline medium compared to the neutral pH conditions. The noticed behaviour can be attributed to the enhanced production of ·OH due to the increased concentration of hydroxide ions in the alkaline medium. A comparable outcome had been observed earlier in the degradation of the RB5 dye and acid blue 40 dye^46^.

#### Effect of dye concentration with all other parameters

**Figure 8:**
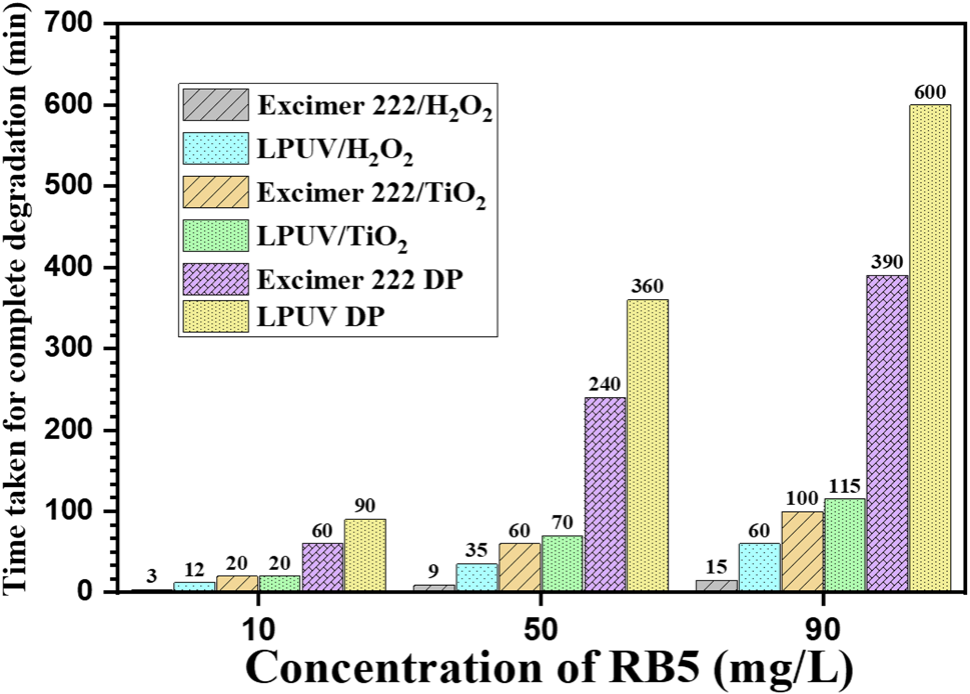
Time comparison of LPUV DP, LPUV/TiO_2_, LPUV/H_2_O_2_, excimer-222 DP, excimer-222/TiO_2_, and excimer-222/H_2_O_2_ for the complete degradation performance of 10 mg/L, 50 mg/L, and 90 mg/L initial RB5 dye concentrations (1 g/L TiO_2_, 10 ppm H_2_O_2_ and pH 10).

The degradation time profiles with the different concentrations of pollutants have also been examined. The degradation process is evaluated by changing the dye concentration from 10 to 90 mg/L at optimized operating parameters and a pH of 10. Figure 8 shows the comparison of LPUV DP, LPUV/TiO_2_, LPUV/H_2_O_2_, excimer-222 DP, excimer-222/TiO_2_, and excimer-222/H_2_O_2_ for the complete degradation performance of 10 mg/L, 50 mg/L, and 90 mg/L initial RB5 dye concentrations. Degradation via DP using LPUV at 254 nm takes a longer time as compared to DP of RB5 dye at 222 nm. This is due to the fact that the molar absorption coefficient and quantum yield of RB5 is higher at 222 nm than at 254 nm. 50 mg/L RB5 dye decomposed fully with a fluence of 2400 mJ/cm^2^, whereas in the case of 90 mg/L it is degraded only $\sim$ 65% at the same UV fluence.

The degradation of RB5 dye (50 mg/L) using a DBD Kr/Cl_2_ excimer source with 1 g/L TiO_2_ requires a duration of 60 min, whereas in the case of LPUV/TiO_2_ it takes 70 min. The addition of TiO_2_ with excimer 222 and LPUV does not show much difference in the degradation because of almost similar absorption coefficient of TiO_2_ at 222 nm and 254 nm. In contrast, when H_2_O_2_ was introduced in the dye suspension and exposed to DBD Kr/Cl_2_ excimer source, it showed exceptional results, and complete degradation took only 9 min, whereas in the case of LPUV/H_2_O_2_ it takes 35 min. The observed behaviour might be attributed to the increased generation of ·OH in excimer-222/H_2_O_2_, ascribed to the higher molar absorption coefficients of H_2_O_2_ at 222 nm. Table 1 shows a comparative analysis of RB5 degradation using various UV/AOP-based reactors operating at different power ratings. It reveals that the dye degradation rate is higher in excimer-222/ H_2_O_2_ compared to the other UV based reactors reported earlier in the literature.

**Table 1:** Comparative analysis of various UV-based reactors for the degradation of RB5 dye.

Pollutant	Type of reactor	Concentration	Time (min)	Power (W)	Degradation efficiency (%)	Electrical energy per order (kWh/m^3^)	References
Acid red 37	UV 254 nm/TiO_2_ (0.5 g/L)	1.0 × 10^–4^ M	T_0.5_ = 117	43	–	714.2	^47^
Malachite green	Ultrasonic (US)	5 mg/L	–	49	–	633.79	^48^
	US/UV/H_2_O_2_ (400 mg/L)			64		19.98	
RhB	Xe lamp/TiO_2_	10 mg/L	60	300	100	–	^49^
MB	UV/TiO_2_ (0.1 g/L)	10 ppm	180	6	97	–	^50^
RB 5	Excimer 222/TiO_2_	50 mg/L	60	15	100	85	This work
RB5	Excimer 222/H_2_O_2_	50 mg/L	9	15	100	13	This work

### EEO and COD Analysis

**Figure 9:**
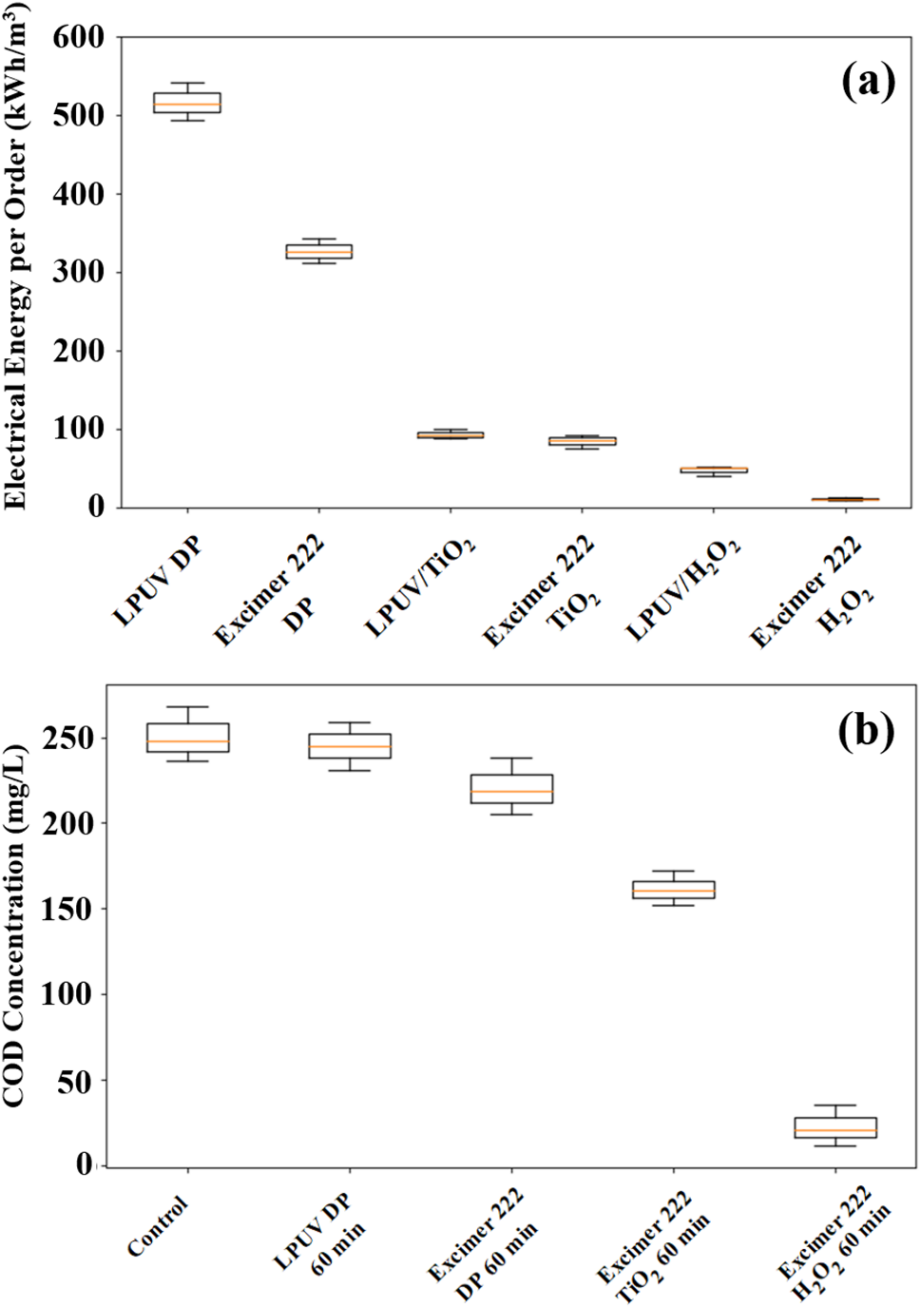
(**a**) Electrical energy per order corresponding to the various treatment methods for complete degradation of RB5 dye (50 mg/L) and (**b**) COD removal as a function of different treatment methods (*C*_0_ = 50 mg/L, 1 g/L TiO_2_, and 10 ppm H_2_O_2_ and pH 10).

When evaluating the potentiality of UV/AOP-based wastewater treatment techniques for commercialization, electrical energy per order (EEO) is an essential indicator. In this study, the EEO of the DBD Kr/Cl_2_ excimer source is computed for different treatment methods using Eq. 5. For the complete degradation of RB5, the EEO in the case of excimer-222/TiO_2_ is found to be 85.6 kWh/m^3^ for 50 mg/L dye concentration and 1 g/L TiO_2_, whereas the EEO in the case of excimer-222/H_2_O_2_ is found to be 12.83 kWh/m^3^ for 50 mg/L dye concentration and 10 ppm H_2_O_2_. Figure 9a shows the EEO of the developed reactor for different treatment methods used in this work. Elbadawy et al.^47^ used TiO_2_ nano photocatalysts with conventional UV-C light (4.0 mW/cm^2^) for the degradation of acid red 37 dye and reported a maximum EEO of 714 kWh/m^3^. The present excimer-UV/H_2_O_2_ system has shown a reasonably lower EEO than many other reported UV/AOP reactors (see Table 1), indicating its potential for industrial exploitation.

Chemical oxygen demand analysis is employed to determine the mineralization of treated wastewater by comparing the untreated and treated wastewater solutions. The COD concentration of untreated dye solution (50 mg/L) and treated with LPUV DP, excimer-222 DP, excimer-222/TiO_2_, and excimer-222/H_2_O_2_ at optimized parameters are shown in Fig. 9b. COD values are measured only after the 60 min of treatment time, to set a better baseline for comparison. It was found that the initial concentration of COD was 248 mg/L, and no significant change in COD concentration was observed when the dye solution was treated with LPUV DP and excimer-222 DP. When treated with excimer-222/TiO_2_, the COD concentration declined to 160.32 mg/L, which corresponds to 35.35% mineralization of RB5 dye. The process of excimer-222/H_2_O_2_ shows some promising results as the rate of mineralization increases up to 91.62% in 60 min of treatment time (as shown in Figure S4). In the case of the excimer-222/H_2_O_2_ process, COD is decreased by 47.7% after a treatment duration of 9 min, when the rate of decolourization of RB5 dye was determined to be 99.9%. Achieving a COD decrease of over 90% will require 60 min, given a dye concentration of 50 mg/L and 10 ppm H_2_O_2_. It has been shown that an increase in treatment time or UV dose is required to achieve more mineralization. Moreover, the RB5 dye suspension treated with an excimer-222/H_2_O_2_ exhibited a notable reduction in COD values, indicating its reactive azo dye degradation ability. The findings show considerable potential for the rapid mineralization of textile wastewater, thereby presenting a viable approach for treating such effluent.

### Degradation Pathway of RB5 dye

**Figure 10:**
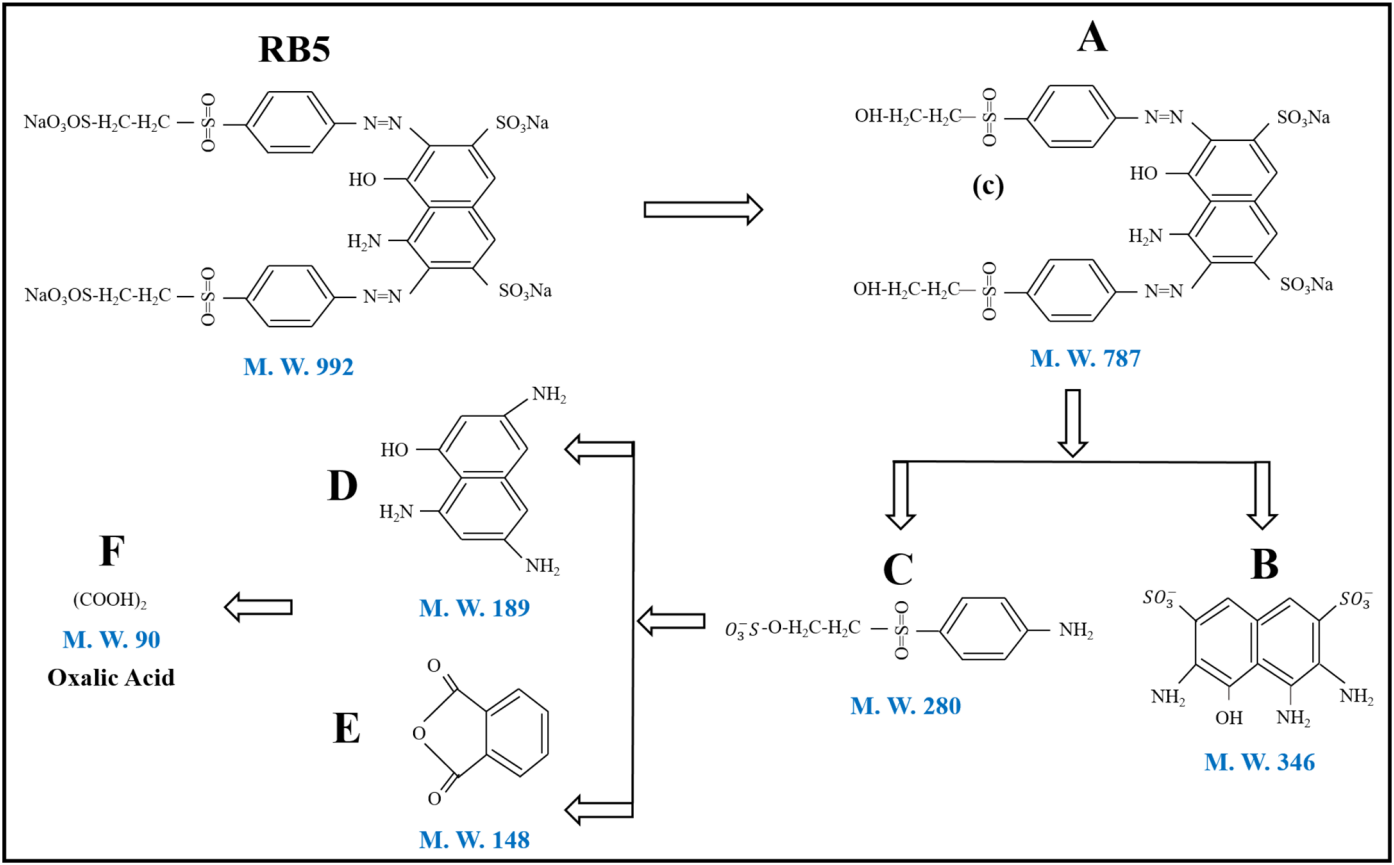
Possible degradation pathway of RB5 dye under excimer-222 assisted photocatalytic mechanism.

The present study investigates the degradation mechanism of RB5 dye by utilising HR-MS and FTIR techniques using untreated and excimer-222/H_2_O_2_ treated wastewater samples. Fragmented by-products are identified through the utilisation of fragmentation data and m/z values obtained through mass spectrometry analysis. A potential mechanism for the degradation of RB5 dye has been proposed and illustrated in Fig. 10, based on the intermediates of its degradation and previous publications^51,52^. The degradation process of RB5 dye involves several sequential processes, including the dissociation of the azo (–N=N–) bond, as observed in FTIR analysis at 1458 cm^−1^ in both untreated and treated samples. Additionally, the functional group undergoes rearrangement, the ring is opened, and the dye undergoes mineralization. Figure S5 displays the FTIR analysis results of untreated and treated (excimer 222/H_2_O_2_) wastewater samples.

The C–O bond in RB5 dye is initially broken due to the direct interaction between the ·OH and organic pollutants, resulting in the formation of an intermediate, depicted as A in Fig. 10. Moreover, the dissociation of the C-SO_3_ link leads to the creation of product D through the fragmentation of product B. Furthermore, fragmentation of different intermediate products results in the formation of oxalic acid through the ring opening mechanism. The benzene rings underwent an ·OH attack, resulting in their fragmentation. Consequently, the RB5 dye underwent degradation into intermediate molecules with lower resilience, ultimately leading to the formation of CO_2_ and H_2_O by mineralization. The degradation of dyes can be inferred to involve the cleavage of azo linkages, leading to the generation of aromatic amines, subsequently resulting in their full disintegration^51,53^. The data analysis revealed that the azo (N=N) bond present in the RB5 dye has undergone degradation, forming many intermediate products.

### Toxicity analysis and reusability of treated wastewater for agriculture purposes

**Figure 11:**
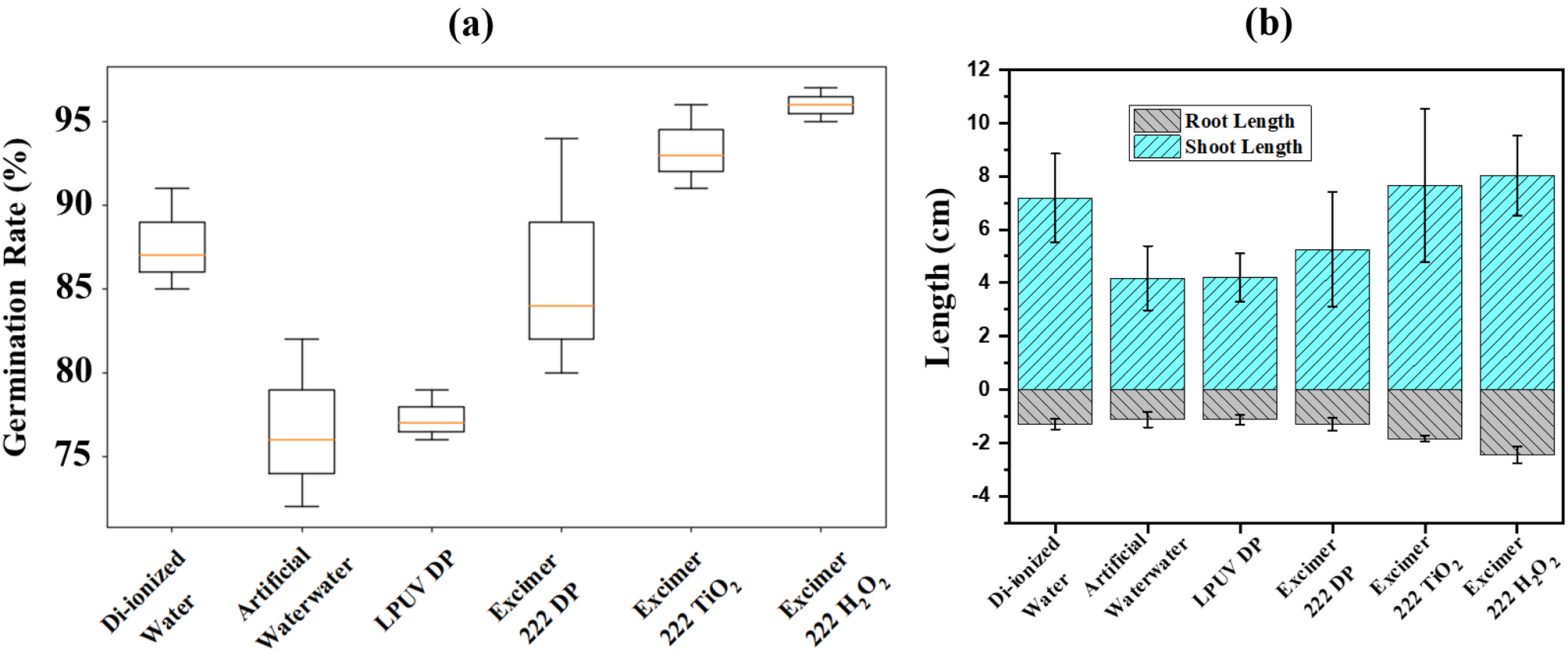
(**a**) Box plot of the Kruskal–Wallis test for values of germination percentage of the Raphanus sativus seeds obtained from different treatment methods, (**b**) shoot and root length of Raphanus seeds after 3 d.

The germination ability of untreated and treated artificial wastewater is assessed by directly sowing Raphanus sativus seeds into the soil. The germination of the Raphanus sativus seeds was carried out using different water samples, including deionized water, artificial wastewater, LPUV DP, excimer-222 DP, excimer-222/TiO_2_ (1 g/L), and excimer-222/H_2_O_2_ (10 ppm) treated dye wastewater. The germination process can occur for three days in a controlled environment. The analysis and comparison of seed germination ability of treated effluent with various methods is conducted by measuring the length of shoots and roots. The germination percentage, root length, and shoot length after a three-day incubation period are summarised in Fig. 11a and b.

The suspension resulting from excimer-222/TiO_2_ and excimer-222/H_2_O_2_ processes shows positive effects on Raphanus sativus seed germination and growth when compared to untreated artificial wastewater, LPUV DP and excimer 222 DP. The enhanced rate of germination was recorded in the excimer-222/H_2_O_2_ process because of the complete mineralization of RB5 dye. The seed germination rate for excimer-222/ H_2_O_2_ treated wastewater is 96%, but for excimer-222/TiO_2_, it is approximately 88% despite the same treatment duration. The excimer-222/TiO_2_ treated wastewater may contain a higher concentration of non-degraded dye and catalyst elements than the excimer-222/H_2_O_2_ treated wastewater. The root and shoot length in dye wastewater is significantly reduced due to nutritional deficiency caused by the high concentration of organic pollutants.

## Discussion

Increasing concentration of organic dyes in textile wastewater has become a global challenge due to their adverse effects on environment such as soil pollution and aquatic life. The ability of UV radiation to decompose organic pollutants has piqued the interest of multidisciplinary researchers. The detrimental consequences of organic dyes on the environment, including soil degradation and aquatic life, have made the concentration of these pigments in textile effluent a global concern. Multidisciplinary researchers are interested to understand to know how UV light breaks down organic contaminants. The present investigation concentrated on the effectiveness of an in-house developed Kr/Cl_2_ far UV-C (222 nm) radiation source on the decomposition of organic dyes dissolved in sea water. The use of H_2_O_2_ as a radical promotor is also tested. Furthermore, by replicating the breakdown of textile effluent, this study evaluated the effects of 222 nm exposure on dye degradation in the presence of varying dye concentrations, catalyst loading, and pH levels.

In the conventional AOPs, the use of 254 nm sources poses a challenging problem because of mercury content, which is even more detrimental to the environment than the treated pollutants. Another concern related to these lamps is their routine replacement and often filament failure^54^. The excimer sources are considered as the best alternative for the conventional UV lamps and proven to be an efficient source for water treatment applications^44,45^. In this study, we have designed and developed a mercury free 222 nm excilamp by using the concept of plasma generation in dielectric barrier discharge (DBD) configurations. It has been observed that the developed DBD Kr/Cl_2_ excilamp has an instant start-up with full radiation output ($\sim$ 2.0 mW/cm^2^), in contrast to the mercury-based UV-C lamp, which has a start-up time of about 2.5 min^55^. The thermal conductivity of the treated suspension due to various reactive species can also be estimated by using numerical modelling techniques^56,57^. The Kr/Cl_2_ excilamp is positioned above the water level, which has produced two important benefits. One benefit is that it prevents the fouling of the excilamp. Another benefit, as described by Rao et al.^58^, is the increased formation of ·OH. This is due to the accelerated photolysis of H_2_O_2_ occurring at the air–water interface rather than in the volume or bulk phase.

The intensity and wavelength of the UV source significantly influence the photocatalytic activity and dye degradation efficiency^59^. This study reveals that for the higher dye concentration, longer treatment time or higher fluence rate is required (see Fig. 8). As the dye concentration increases, the number of excimer UV-C (222 nm) photons that can reach the surface of the photocatalyst decreases, leading to a decrease in the formation of ·OH^59^. Some studies also suggest that degradation/mineralization of suspension occurs by direct transfer of electrons and holes from the TiO_2_ surface to the dye molecules^46,60^ as,

9 $$Dye + e_{cb}^{ - } \to Dye^{ \cdot - } \to Degradation\;Products$$

10 $$Dye + h_{vb}^{ + } \to Dye^{ \cdot - } \to Degradation\;Products$$

From Eqs. (9) and (10), it is clear that there is a negative correlation between dye concentration and photocatalyst activation. Specifically, as the dye concentration increases, the availability of electrons for direct reduction and holes for direct oxidation of the dye molecule decreases.

The increase in catalyst loading in the dye suspension increases the rate of degradation because the intensity of 222 nm radiation absorbed by TiO_2_ increases with increasing TiO_2_ dosage in the suspension. Interestingly, when the TiO_2_ dose exceeded 1 to 1.25 g/L, the degradation rate constant declined. This is due to the fact that an increased concentration of TiO_2_ in the suspension increases the reflectance of far UV-C light, which is mostly responsible for the decline in the RB5 dye degradation efficiency, as reported elsewhere^61^. Despite of having a reasonable degradation efficiency, TiO_2_ photocatalysis has two main disadvantages: a high rate of recombination of electron-hole pair and the need for additional process of photocatalysts removal from the treated wastewater that is used in nano-powder form, which increases the overall cost of the treatment. The utilization of catalyst in wastewater suspensions is constrained by issues such as particle aggregation, slurry formation, and the expenses associated with catalyst separation. Thus, to address these constraints, magnetically retrievable catalysts and catalytic immobilization in the form of films or coatings on stationary supports have been employed^62^. The use of this method on a large scale can be limited by the need to replace the immobilized catalysts after certain cycles, as well as by mass transfer limits on the immobilized catalysts and the low quantum yield for ·OH radical generation^63^.

This study has further utilized H_2_O_2_ as a radical oxidant in the wastewater suspension. In the case of excimer-222/TiO_2_, time required for the complete degradation of RB5 was 60 min, but in the case of excimer-222/H_2_O_2_, the time required for the degradation of RB5 is only 10 min. These exceptional results obtained in the case of H_2_O_2_ addition are due to the powerful oxidizing nature of H_2_O_2_, reacting rapidly with highly energetic photons of far UV-C light to produce ·OH through the following reaction^46^,

11 $$H_{2} O_{2} + hv \left( {222\;{\text{nm}}} \right) \to 2 \cdot OH$$

In addition, the background water matrix plays significant role in case of 222 nm radiation. Nitrates presents in wastewater absorbs light extensively at 222 nm and produces ·OH at the quantum yields of 9–21% by the process as mentioned in Eq. (10)^64^ Excimer-222/H_2_O_2_ process dominates in wastewater degradation and mineralization because of the following interactions:

12 $$NO_{3}^{ - }\xrightarrow{{hv\left( {222\;{\text{nm}}} \right)}} \cdot NO_{2} + \cdot O^{ - }$$

13 $$\cdot O^{ - } + H_{2} O \to \cdot OH + OH^{ - }$$

The addition of TiO_2_ and H_2_O_2_ together reduces the degradation efficiency because H_2_O_2_ scavenges the photo-generated oxidizing species, i.e., ·OH and ${h}_{vb}^{+}$ (see Eqs. 14–16)^65^ would otherwise be available for the oxidative destruction of the dye molecules. Excimer-222/H_2_O_2_ is regarded as a viable approach for wastewater treatment due to its tendency to generate ·OH and its sludge-free operation, characterised by accelerated kinetics^66^.

14 $$H_{2} O_{2} + e^{ - } \to \cdot OH + OH^{ - }$$

15 $$H_{2} O_{2} + \cdot OH \to H_{2} O + HO_{2}^{ \cdot }$$

16 $$H_{2} O_{2} + 2h_{vb}^{ + } \to O_{2} + 2H^{ + }$$

17 $$RB5 + \cdot OH \to CO_{2} + H_{2} O + Byproducts$$

This study reveals that the degradation of dye is pH sensitive and the highest rate of degradation has been found in the alkaline medium (pH 10) than the acidic and neutral medium. The higher production of hydroxyl radical in the alkaline medium could have increased the reaction rate. In general, the pH of the textile effluents is found alkaline ($\sim$ 10 ± 1)^67^ and the obtained results hold a good opportunity to degrade the textile effluents in a more effective way without the need of the neutralization.

Electrical energy per order (EEO) and COD tests are conducted for several process combinations (LPUV DP, Excimer 222 DP, LPUV/TiO_2_, Excimer-222/ TiO_2_, LPUV/H_2_O_2_, Excimer-222/ H_2_O_2_), in addition to dye degradation. The optimized values, at which the highest percentage degradation (99.9%,) and COD removal (91.62%) was attained with low EEO of 12.84 kWh/m^3^ is: H_2_O_2_ concentration—10 ppm, UV 222 intensity—1.054 mW/cm^2^, and pH—10. The transformation of organic compounds into inorganic compounds directly impacts the reduction of COD and is directly related to the breakdown of organic matter^7^. The decrease in COD value is basically due to the excessive presence of ·OH, and due to its higher oxidation potential (2.8 V), a higher degree of mineralization has been achieved^68^.

**Figure 12:**
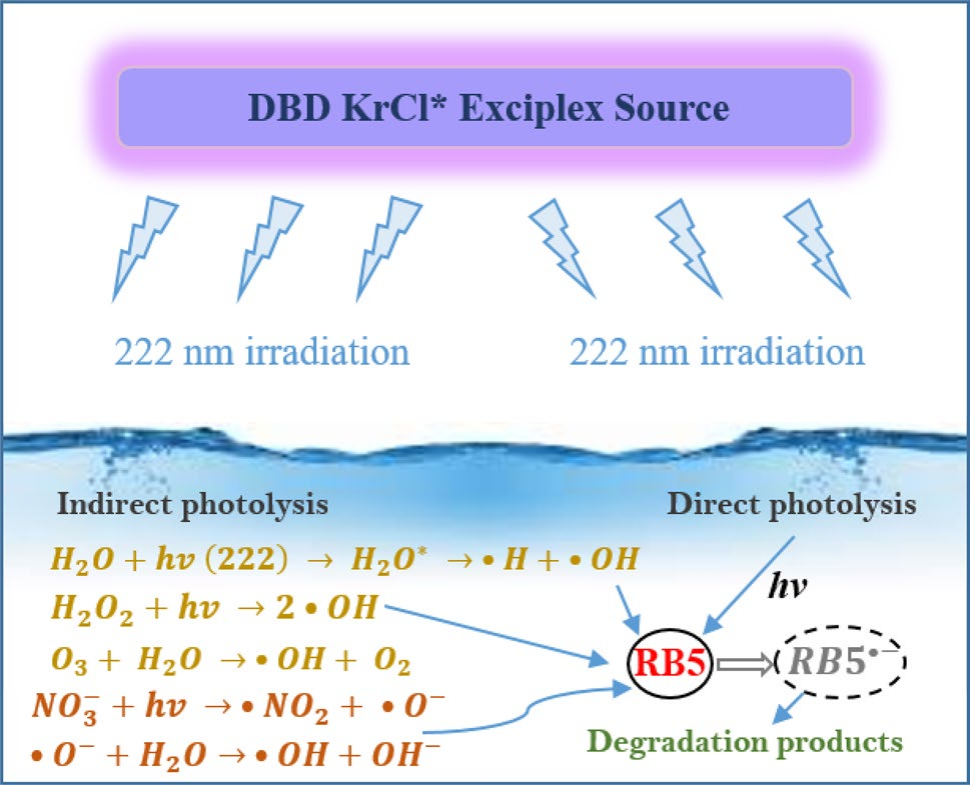
Proposed reaction mechanism of RB5 degradation with 222 nm excimer source.

Understanding how various reactive species and 222 nm radiation cause degradation is essential for process standardization. The ·OH is the major dye degrading agent and there are many ways to generate ·OH, e.g., by the decomposition of H_2_O_2_ under excimer 222 exposure, and by the reaction of ozone and/or UV 222 light with the water molecules. The proposed reaction mechanism of RB5 degradation with 222 excilamp is shown in Fig. 12 and briefly described as follows:

1. Formation of ·OH by the action of UV 222 light with H_2_O_2_ and water molecules (Eq. 11) (most probable);
2. Formation of ·OH by the photolysis of nitrates (already presents in the wastewater matrix) under 222 nm (Eq. 12 and 13).
3. Degradation of dye molecules with the direct photolysis with 222 nm photon due to high quantum yield of RB5 at 222 nm.
4. Reaction of dissolved ozone with the water molecules (least probable);
5. At last, degradation and mineralization of dye molecules via the oxidation of ·OH (Eq. 17).

The findings indicate the potential use of DBD Kr/Cl_2_ excilamp in the context of wastewater treatment and its possible application in agriculture. Additional research is required to investigate the mechanisms responsible for these outcomes to improve the treatment method for broader use.

### Industrial relevance

The developed process holds good promises for its use in the industrial wastewater treatment. Several tangible applications demonstrating the significance of research undertaken include the followings:

1. *Wastewater treatment* the use of Kr/Cl_2_ excilamp can be an efficient way to clean wastewater that contains dyes from different industries, such as the production of paper, leather, and textiles. By aiding in the degradation and breakdown of dyes, this method can improve the safety of water for discharge into the environment or later reuse.
2. *Environmental remediation* the use of Kr/Cl_2_ excilamp can reduce contamination in the environment caused by dye spills or improper disposal practices. An important issue that requires attention is mercury discharge into the open environment, which can be prevented with the use of mercury-free Kr/Cl_2_ excilamp.
3. *Textile industry* integrating Kr/Cl_2_ excilamp into textile manufacturing processes can serve as a means to purify wastewater before its release. This can reduce the ecological effect of textile manufacture and can ensure compliance with environmental regulations.

## Conclusion

A DBD-based far UV-C (222 nm) Kr/Cl_2_ excilamp has been designed and developed. The advantage of the developed source is demonstrated for the degradation of complex organic molecules like RB5 dye, in DP and in AOP processes using TiO_2_/H_2_O_2_. The exceptional performance of the developed excilamp in the degradation of RB5 dye is achieved due to the higher molar absorption coefficient of RB5 dye at 222 nm than 254 nm. The quantum yield of RB5 dye at excimer-222/H_2_O_2_ is found 31 times higher as compared to excimer-222/TiO_2,_ demonstrating the effectiveness of excimer-222/H_2_O_2_ over other processes. It is found that the degradation rate of RB5 is $\sim$ 27 times faster in excimer-222/H_2_O_2_ process, whereas in excimer-222/TiO_2_ it is only 4 times faster as compared to excimer-222 DP. The order of degradation/mineralization is found to be excimer-222/H_2_O_2_ > LPUV/H_2_O_2_ > excimer-222/TiO_2_ > LPUV/TiO_2_ > excimer-222 DP > LPUV DP. The EEO of the excimer-222/H_2_O_2_ process is $\sim$ 500% higher than the excimer-222/TiO_2_ process. The process that has been developed is environmentally friendly due to the absence of mercury usage, and it effectively removes the need for catalyst recovery from the treated effluent following degradation. The water resulting from these different treatments has been used for the germination of Raphanus sativus seed and it has shown a positive impact on the germination percentage, root length, and shoot length than untreated wastewater, demonstrating its non-toxic nature. The increased operation flexibility with lower EEO makes this technology a potential alternative to conventional LPUV-based AOPs for commercial exploitation. To make sure the reactor works for industrial purposes, more investigation and thorough pilot scale testing of the design are required. More research on the use of far UV-C excimer sources for sustainable agricultural techniques is also necessary.

### Supplementary Information

Supplementary Information.

## Data Availability

The datasets generated during and/or analyzed during the current study are available from the corresponding author on reasonable request.

## References

[1] Al-Tohamy R (2022). A critical review on the treatment of dye-containing wastewater: Ecotoxicological and health concerns of textile dyes and possible remediation approaches for environmental safety. Ecotoxicol. Environ. Saf..

[2] Goswami R (2024). Nanocellulose: A comprehensive review investigating its potential as an innovative material for water remediation. Int. J. Biol. Macromol..

[3] Khan NA (2023). Emerging membrane technology and hybrid treatment systems for the removal of micropollutants from wastewater. Desalination.

[4] Ceretta MB, Nercessian D, Wolski EA (2021). Current trends on role of biological treatment in integrated treatment technologies of textile wastewater. Front. Microbiol..

[5] Zhang Y, Shaad K, Vollmer D, Ma C (2021). Treatment of textile wastewater by advanced oxidation processes—A review. Glob. Nest J..

[6] Vishnu G (2023). Photodegradation of methylene blue dye using light driven photocatalyst-green cobalt doped cadmium ferrite nanoparticles as antibacterial agents. J. Clean. Prod..

[7] Deng Y, Zhao R (2015). Advanced oxidation processes (AOPs) in wastewater treatment. Curr. Pollut. Reports.

[8] Xu J, Huang CH (2023). Enhanced direct photolysis of organic micropollutants by far-UVC light at 222 nm from KrCl∗ excilamps. Environ. Sci. Technol. Lett..

[9] Ahlawat K (2023). Analysis of a UV photocatalytic oxidation-based disinfection system for hydroxyl radicals, negative air ions generation and their impact on inactivation of pathogenic micro-organisms. Rev. Sci. Instrum..

[10] Huang Y (2018). Efficient degradation of cytotoxic contaminants of emerging concern by UV/H_2_O_2_. Environ. Sci. Water Res. Technol..

[11] Al-Mamun MR, Kader S, Islam MS, Khan MZH (2019). Photocatalytic activity improvement and application of UV-TiO_2_ photocatalysis in textile wastewater treatment: A review. J. Environ. Chem. Eng..

[12] Lee SY, Park SJ (2013). TiO_2_ photocatalyst for water treatment applications. J. Ind. Eng. Chem..

[13] Pavel M (2023). Photocatalytic degradation of organic and inorganic pollutants to harmless end products: Assessment of practical application potential for water and air cleaning. Catalysts.

[14] Fadheela AS, Ali AR, Hawraa AS, Layla H, Safa T (2020). Toxicity evaluation of TiO_2_ nanoparticles embedded in toothpaste products. GSC Biol. Pharm. Sci..

[15] Bar-Niv N (2022). Advanced oxidation process UV-H_2_O_2_ combined with biological treatment for the removal and detoxification of phenol. J. Water Process Eng..

[16] Cédat B, de Brauer C, Métivier H, Dumont N, Tutundjan R (2016). Are UV photolysis and UV/H_2_O_2_ process efficient to treat estrogens in waters? Chemical and biological assessment at pilot scale. Water Res..

[17] Yin R, Anderson CE, Zhao J, Boehm AB, Mitch WA (2023). Controlling contaminants using a far-UVC-based advanced oxidation process for potable reuse. Nat. Water.

[18] Matafonova G, Batoev V (2012). Recent progress on application of UV excilamps for degradation of organic pollutants and microbial inactivation. Chemosphere.

[19] Payne EM, Liu B, Mullen L, Linden KG (2022). UV 222 nm emission from KrCl∗ excimer lamps greatly improves advanced oxidation performance in water treatment. Environ. Sci. Technol. Lett..

[20] Aristizábal A, Perilla G, Lara-Borrero JA, Diez R (2020). KrCl and XeCl excilamps and LP-Hg lamp for UV and UV/H_2_O_2_ decolourization of dyes in water. Environ. Technol. (United Kingdom).

[21] Yamano N (2020). Long-term effects of 222-nm ultraviolet radiation C sterilizing lamps on mice susceptible to ultraviolet radiation. Photochem. Photobiol..

[22] Cadet J (2020). Harmless effects of sterilizing 222-nm far-UV radiation on mouse skin and eye tissues. Photochem. Photobiol..

[23] Murcia MD, Gómez M, Gómez E, Gómez JL, Christofi N (2011). Photodegradation of congo red using XeBr, KrCl and Cl2 barrier discharge excilamps: A kinetics study. Desalination.

[24] Murcia MD (2020). Comparison of two excilamps and two reactor configurations in the UV-H_2_O_2_ removal process of amaranth. J. Water Process Eng..

[25] Gan J (2023). Degradation and dechlorination of trichloroacetic acid induced by an in situ 222 nm KrCl* excimer radiation. Chemosphere.

[26] Li T, Zhang Y, Gan J, Yu X, Wang L (2023). Superiority of UV222 radiation by in situ aquatic electrode KrCl excimer in disinfecting waterborne pathogens: Mechanism and efficacy. J. Hazard. Mater..

[27] Ahlawat K, Jangra R, Ish A, Jain N, Prakash R (2024). A dielectric barrier discharge based low pressure narrow band far UV-C 222 nm excimer lamp and its efficiency analysis. Phys. Scr..

[28] Bolton JR, Linden KG (2003). Standardization of methods for fluence (UV Dose) determination in bench-scale UV experiments. J. Environ. Eng..

[29] Wang X (2014). The influence of crystallite size and crystallinity of anatase nanoparticles on the photo-degradation of phenol. J. Catal..

[30] Jangra R, Ahlawat K, Dixit A, Prakash R (2023). Efficient deactivation of aerosolized pathogens using a dielectric barrier discharge based cold - plasma detergent in environment device for good indoor air quality. Sci. Rep..

[31] Ahlawat K (2022). Photocatalytic oxidation conveyor ‘PCOC’ system for large scale surface disinfection. Rev. Sci. Instrum..

[32] Jangra R, Ahlawat K, Prakash R (2023). An SDBD plasma-based source for efficient degradation of VOCs in an enclosed environment. Phys. Lett. Sect. A Gen. At. Solid State Phys..

[33] Makuła P, Pacia M, Macyk W (2018). How to correctly determine the band gap energy of modified semiconductor photocatalysts based on UV–vis spectra. J. Phys. Chem. Lett..

[34] Sun D, Zhang X, Du H, Fang L, Jiang P (2017). Application of liquid organic salt to cotton dyeing process with reactive dyes. Fibers Polym..

[35] Awais M, Salahuddin T (2024). Radiative magnetodydrodynamic cross fluid thermophysical model passing on parabola surface with activation energy. Ain Shams Eng. J..

[36] Liu B, Mullen L, Payne EM, Linden KG (2023). Accelerated ultraviolet treatment of carbamazepine and NDMA in water under 222 nm irradiation. Environ. Sci. Technol..

[37] Pandey S (2023). Selective generation of nitrate and nitrite in plasma activated water and its physicochemical parameters analysis. Phys. Lett. A.

[38] Saud HR, Al-Taweel SS (2016). New route for synthesis of pure anatase TiO_2_ nanoparticles via utrasound-assisted sol-gel method. J. Chem. Pharm. Res..

[39] Reddy KM, Manorama SV, Reddy AR (2003). Bandgap studies on anatase titanium dioxide nanoparticles. Mater. Chem. Phys..

[40] Pawar M, Sendoǧdular ST, Gouma P (2018). A brief overview of TiO_2_ photocatalyst for organic dye remediation: Case study of reaction mechanisms involved in Ce-TiO_2_ photocatalysts system. J. Nanomater..

[41] Khan I, Saeed K, Khan I (2019). Nanoparticles: Properties, applications and toxicities. Arab. J. Chem..

[42] Cottre T (2021). Interaction of water with atomic layer deposited titanium dioxide on p-Si photocathode: Modeling of photoelectrochemical interfaces in ultrahigh vacuum with cryo-photoelectron spectroscopy. Adv. Mater. Interfaces.

[43] Kritikos DE, Xekoukoulotakis NP, Psillakis E, Mantzavinos D (2007). Photocatalytic degradation of reactive black 5 in aqueous solutions: Effect of operating conditions and coupling with ultrasound irradiation. Water Res..

[44] Li D (2017). Effect of advanced oxidation on N-nitrosodimethylamine (NDMA) formation and microbial ecology during pilot-scale biological activated carbon filtration. Water Res..

[45] Wang C, Moore N, Bircher K, Andrews S, Hofmann R (2019). Full-scale comparison of UV/H_2_O_2_ and UV/Cl_2_ advanced oxidation: The degradation of micropollutant surrogates and the formation of disinfection byproducts. Water Res..

[46] Tang C, Chen V (2004). The photocatalytic degradation of reactive black 5 using TiO_2_/UV in an annular photoreactor. Water Res..

[47] Elbadawy HA, Elhusseiny AF, Hussein SM, Sadik WA (2023). Sustainable and energy-efficient photocatalytic degradation of textile dye assisted by ecofriendly synthesized silver nanoparticles. Sci. Rep..

[48] Behnajady MA, Vahid B, Modirshahla N, Shokri M (2009). Evaluation of electrical energy per order (EEO) with kinetic modeling on the removal of malachite green by us/UV/H_2_O_2_ process. Desalination.

[49] Li X (2023). Synergistic effect of Y doping and reduction of TiO_2_ on the improvement of photocatalytic performance. Nanomaterials.

[50] Azeez F (2018). The effect of surface charge on photocatalytic degradation of methylene blue dye using chargeable titania nanoparticles. Sci. Rep..

[51] Bilal M (2018). Toxicological assessment and UV/TiO_2_-based induced degradation profile of reactive black 5 dye. Environ. Manag..

[52] Plum A, Braun G, Rehorek A (2003). Process monitoring of anaerobic azo dye degradation by high-performance liquid chromatography-diode array detection continuously coupled to membrane filtration sampling modules. J. Chromatogr. A.

[53] El M, Salah W, Din E (2016). Biodegradation of reactive black 5 by Aeromonas hydrophila strain isolated from dye-contaminated textile wastewater. Sustain. Environ. Res..

[54] Borchers, H., Fuller, A. & Malley, J. P. *Assessing the Risk of Mercury in Drinking Water after UV Lamp Breaks*. (2008).

[55] Chatterley C, Linden K (2010). Demonstration and evaluation of germicidal UV-LEDs for point-of-use water disinfection. J. Water Health.

[56] Khan M (2023). Calculating the entropy generation of a Bingham plastic fluid flow due to a heated rotating disk. Int. Commun. Heat Mass Transf..

[57] Malik MY (2016). Mixed convection dissipative viscous fluid flow over a rotating cone by way of variable viscosity and thermal conductivity. Results Phys..

[58] Rao Z (2023). Accelerated photolysis of H_2_O_2_ at the air–water interface of a microdroplet. J. Am. Chem. Soc..

[59] Reza KM, Kurny A, Gulshan F (2017). Parameters affecting the photocatalytic degradation of dyes using TiO_2_: A review. Appl. Water Sci..

[60] Escombe AR (2009). Upper-room ultraviolet light and negative air ionization to prevent tuberculosis transmissio. PLoS Med..

[61] Muruganandham M, Sobana N, Swaminathan M (2006). Solar assisted photocatalytic and photochemical degradation of Reactive Black 5. J. Hazard. Mater..

[62] Lima MJ (2017). Homogeneous and heterogeneous photo-Fenton degradation of antibiotics using an innovative static mixer photoreactor. Chem. Eng. J..

[63] Antonopoulou M, Kosma C, Albanis T, Konstantinou I (2021). An overview of homogeneous and heterogeneous photocatalysis applications for the removal of pharmaceutical compounds from real or synthetic hospital wastewaters under lab or pilot scale. Sci. Total Environ..

[64] Keen OS, Love NG, Linden KG (2012). The role of effluent nitrate in trace organic chemical oxidation during UV disinfection. Water Res..

[65] Ahlawat K, Jangra R, Prakash R (2024). Environmentally friendly UV-C excimer light source with advanced oxidation process for rapid mineralization of azo dye in wastewater. ACS Omega.

[66] Luo M, Xu W, Jeong T (2021). Development and numerical modelling of a novel UV/H_2_O_2_ rotating flow reactor for water treatment. Water Sci. Technol..

[67] Rai PK, Kant V, Sharma RK, Gupta A (2023). Process optimization for textile industry-based wastewater treatment via ultrasonic-assisted electrochemical processing. Eng. Appl. Artif. Intell..

[68] Thanavel M (2019). Combined biological and advanced oxidation process for decolorization of textile dyes. SN Appl. Sci..

