# Supplementary material for: Accelerated mineralization of textile wastewater under 222 nm irradiation from Kr/Cl2 excilamp: an environmentally friendly and energy efficient approach
Source: Sci Rep. 2024 May 31;14:12560. doi: 10.1038/s41598-024-63012-z (PMC11143330; doi:10.1038/s41598-024-63012-z)
Supplement: Supplementary file 1 — Supplementary Information. [file 41598_2024_63012_MOESM1_ESM.docx]

**Supplementary Information**

**Accelerated mineralization of textile wastewater under 222 nm irradiation from Kr/Cl_2_ excilamp: An environmentally friendly and energy efficient approach**

Kiran Ahlawat^1^, Ramavtar Jangra^1^, and Ram Prakash^1,*^

^1^Department of Physics, Indian Institute of Technology Jodhpur, Rajasthan, India, 342037

*Author to whom correspondence should be addressed: [ramprakash@iitj.ac.in](mailto:ramprakash@iitj.ac.in)

***Table S1:*** *Physicochemical properties and structure of RB5 dye*

| **Pollutant** | **CAS number** | **Chemical Structure** | **Formula** | **Molecular weight (g/mol)** |
| --- | --- | --- | --- | --- |
| Reactive Black 5 | 17095-24-8 | 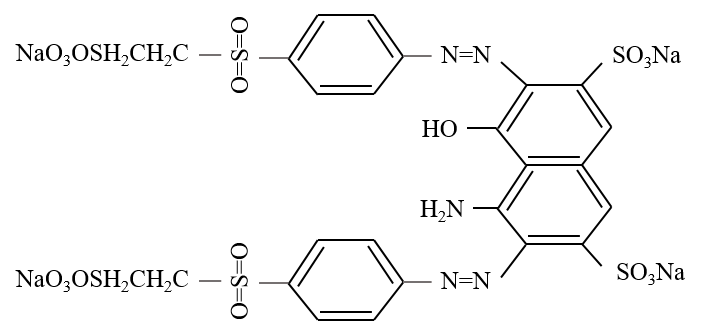 | C_26_H_21_N_5_Na_4_O_19_S_6_ | 991.82 |

**1.1 Materials**

Different-sized quartz tubes were purchased from QSIL GmbH (Germany). Reactive Black 5 (C_26_H_21_N_5_Na_4_O_19_S_6_) dye was purchased from Sigma Aldrich and used as it is without any further processing. The structure and physicochemical properties of RB5 dye are summarized in **Table S1**. H_2_O_2_ (30% w/v), NaOH (97.0%), and H_2_SO_4_ (97.0%) were purchased from Sigma Aldrich. Research grade Krypton balanced with 1% Chlorine (99.99%) was procured from Alchemie Gases and Chemicals Pvt Ltd, India. Argon gas (99.999%) was procured from Scientific and General Agencies, India. Titanium Dioxide (TiO_2_) nanoparticles were synthesized in the laboratory. All of the stock solutions were prepared in artificial wastewater. A conventional LPUV lamp was purchased from Phillips, India. Mercury-free far UV-C Kr/Cl_2_ (222 nm) excilamp was developed using standard laboratory practices as described in the next section ^1^.

**1.2 Development and Process of Kr/Cl_2_ excimer source**

The developed DBD-based far UV-C (222 nm) excimer light source is shown in **Figure 1**. The source consisted of two quartz tubes (wall thickness 1.5 mm) fused co-axially at both ends with a gas gap of 1.5 mm. The inner and outer tube dimensions were 10 mm OD and 7 mm ID, and 16 mm OD and 13 mm ID, respectively, and the lamp length was 160 mm. A wire mesh of stainless steel having a thickness of 0.1 mm was used on the outer surface of the larger OD quartz tube which acts as the ground electrode. A helical-shaped copper wire inserted inside the smaller quartz tube acts as a high-voltage electrode in the co-axial DBD configuration. This electrode was hermetically sealed for electrical isolation. The development of DBD based Kr/Cl_2_ excilamp consisted of multiple parts. Firstly, the gas gap was evacuated up to 1×10^-5^ mbar of base pressure by using a roughing vacuum pump and the turbo molecular pump. After evacuating, the vacuum system was flushed with Argon ten times in a controlled manner to decrease the impurities up to a relatively low level. The gas gap was again evacuated up to 2×10^-6^ mbar pressure and filled with a mixture of research-grade KrCl gas (99.99%) by using the needle valves. The Kr/Cl_2_ gas pressure of 140 mbar was optimized in the experiment and finally, the excilamp was pinched for further experimentations.

The developed excimer source was operated by a bipolar pulsed power supply [1-10 kV, 5-40 kHz, 1 A, 2 µsec pulse width] at different power settings. For the visualization of voltage and current waveforms, a high voltage probe (Tektronix P6015A, 1000:1) and a Rogowski coil (Pearson 110, 0.1 V/A, 20 ns rise time) were connected to a four-channel mixed domain oscilloscope (Tektronix MDO3014, bandwidth 100 MHz, 2.5 GS/s). A calibrated thermocouple and IR camera were used to measure the temperature of the excimer source during experimentation.

The Kr/Cl_2_ excimer can be formed by two mechanisms:

1. Three-body ion-ion recombination reaction,

When the electric field is applied between the electrodes of DBD excilamp, the discharge of gases takes place below atmospheric pressure, triggering the generation of highly energetic electrons, which cause the ionization and excitation of krypton and chlorine molecules. The cations of krypton and anions of chlorine get involved in a three-body recombination reaction with an atom/molecule of active species or buffer gas (M) and form the excimer (KrCl*) as,

$$\mathrm{Kr}^{+}+ \mathrm{Cl}^{-}+M \to\mathrm{KrCl}^{*}+M (R1)$$

1. A harpoon reaction, i.e., a two-body reaction.

An excimer can also be formed by a harpooning reaction in which the excited krypton transfer its loosely bound electron to the chlorine molecule to form an electronically excited state of KrCl*.

$$\mathrm{Kr}^{*}+\mathrm{Cl}_{2} \to\mathrm{KrCl}^{*}+ Cl (R2)$$

Generally, the KrCl* excimer have a short lifetime and decomposes rapidly in the nanosecond timescale and emits photons having a wavelength of 222 nm as

$$\mathrm{KrCl}^{*} \to Kr+Cl+hv \left( 222 nm \right) (R3)$$

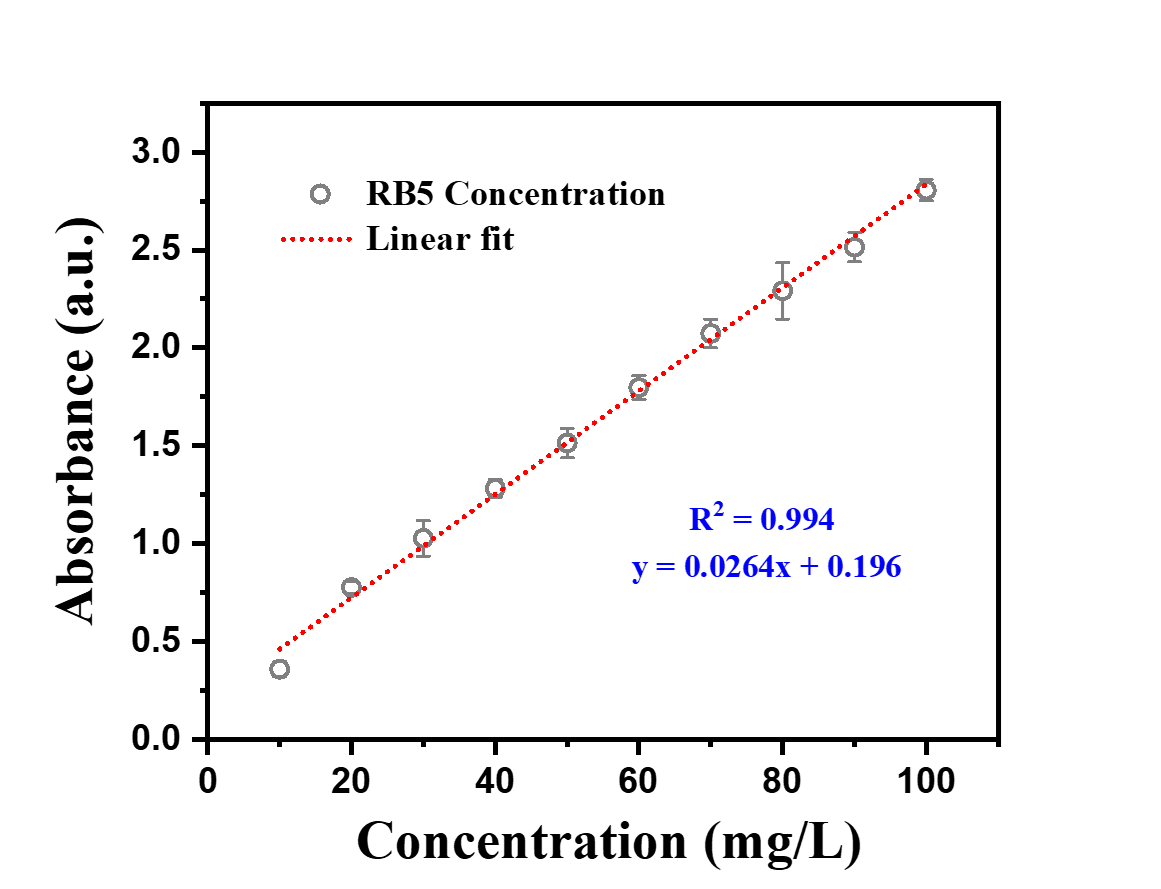


**Figure S1:** Calibration curve of RB5 dye.

**1.3 Reaction mechanism of TiO_2_ photocatalyst**

When TiO_2_ is illuminated with greater energy than the band gap, an electron will move from the valence band to the conduction band to produce holes in the valence band and the electrons in the conduction band.

$${TiO}_{2}+hv \to e_{cb}^{-}+ h_{vb}^{+}$$

In a heterogeneous photocatalysis mechanism, two reaction steps happens successfully:

1. The reduction of oxygen (O_2_) by the electrons, and hydroxyl radicals (•OH) are generated via a series of reaction, as follows:

$$e_{cb}^{-}+ O_{2} \to O_{2}^{\bullet-}$$

$$O_{2}^{\bullet-}+ H^{+} \to{HO}_{2}^{\bullet}$$

$${HO}_{2}^{\bullet}+ {HO}_{2}^{\bullet} \to H_{2}O_{2}+ O_{2}$$

$$H_{2}O_{2}+ e_{cb}^{-} \to\bullet OH+ {OH}^{-}$$

$$H_{2}O_{2}+hv \to2\bullet OH$$

2. The holes metamorphose the water molecules into •OH.

$$h_{vb}^{+}+ H_{2}O \to\bullet OH+ H^{+}$$

These •OH acts as an oxidizing agent and changes toxic organic compounds – principally those incorporated in wastewater – into carbon dioxide, water and other innocuous by-products.

$$Organic Compounds+ \bullet OH \to{CO}_{2}+ H_{2}O$$

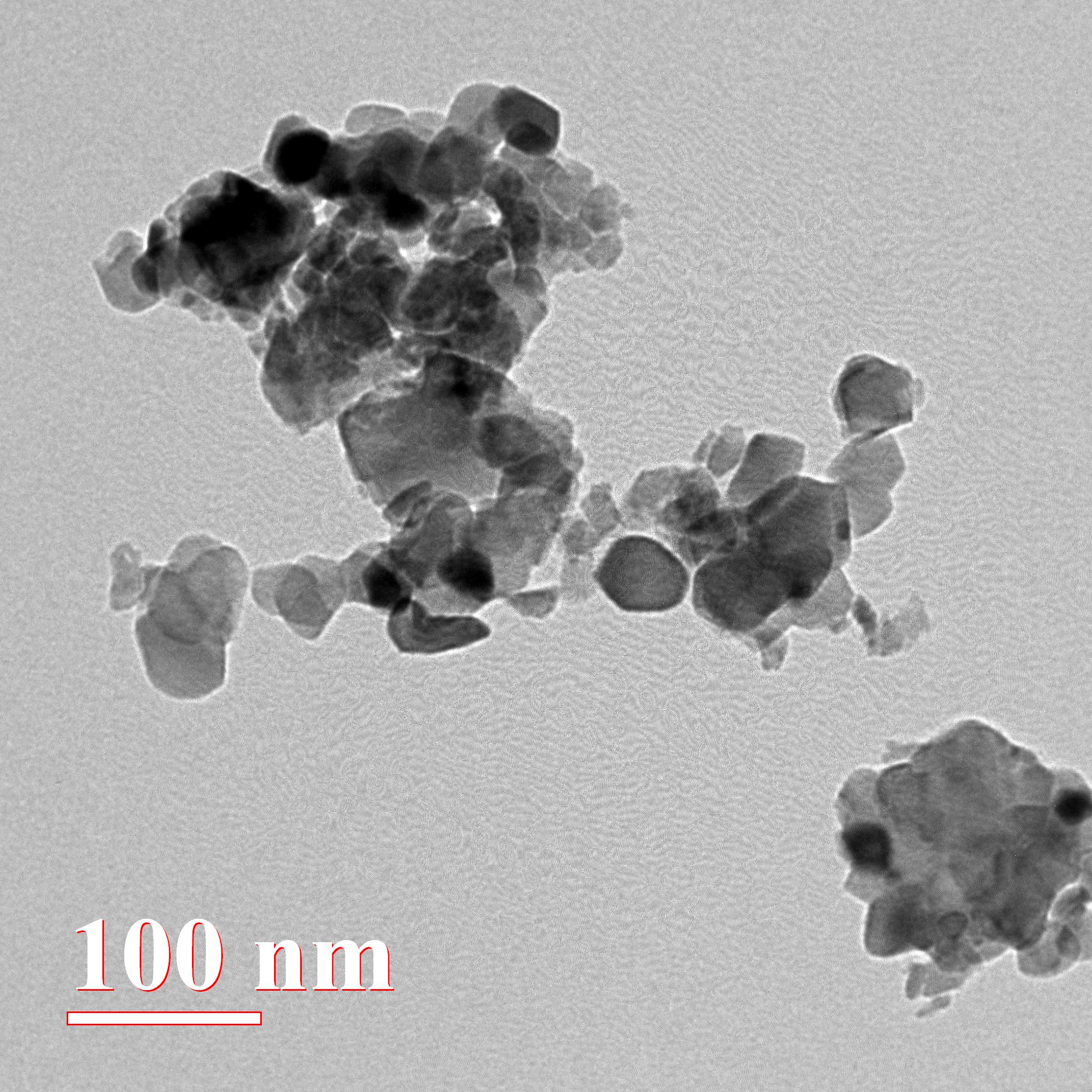


**Figure S2:** HRTEM image of synthesized TiO_2_ nanoparticles.

***Table S2:*** *Molar absorption coefficient and quantum yield of RB5 dye at different wavelengths*.

| **Wavelength/parameters** | **Molar absorption coefficient (M^-1^cm^-1^)** | **Quantum yield** |
| --- | --- | --- |
| 222 nm | 21382 | 0.0502 |
| 254 nm | 16870 | 0.0018 |


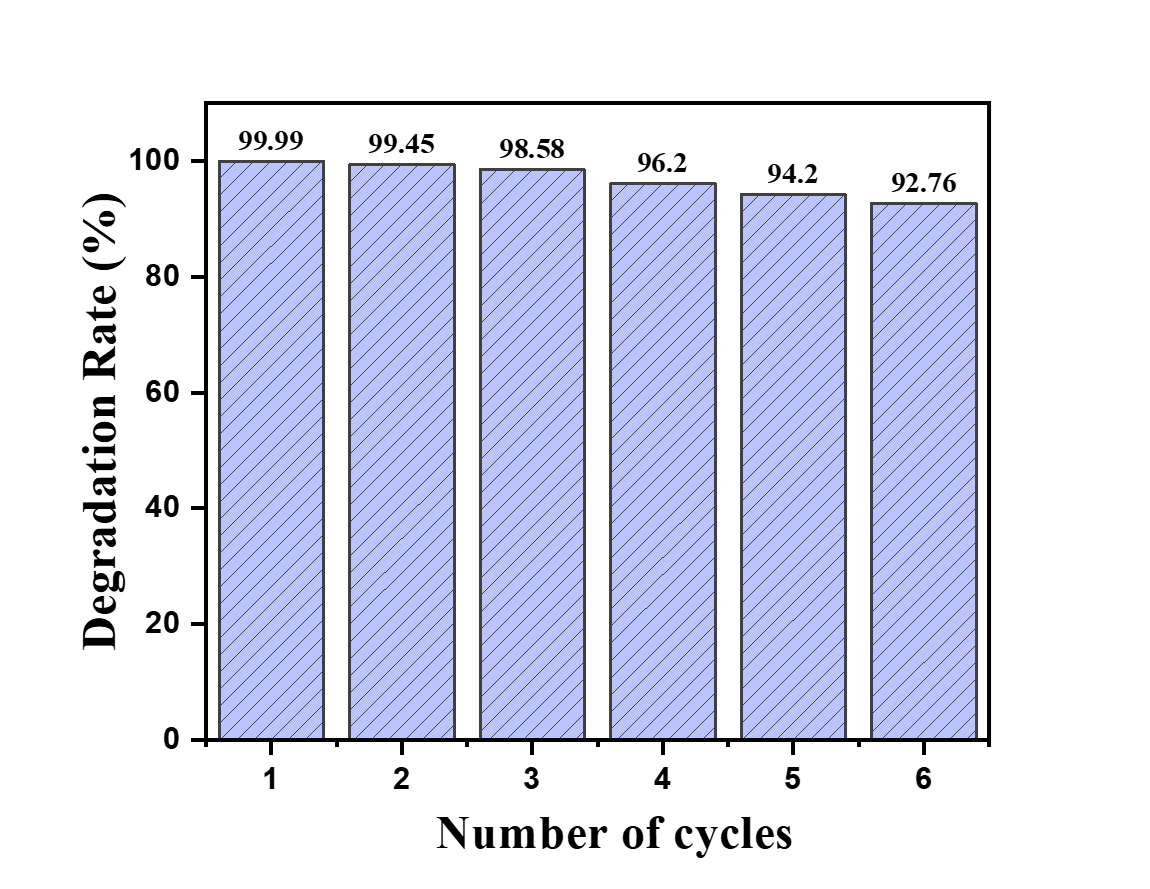


**Figure S3:** Reusability and stability of the synthesized catalyst for up to six cycles.


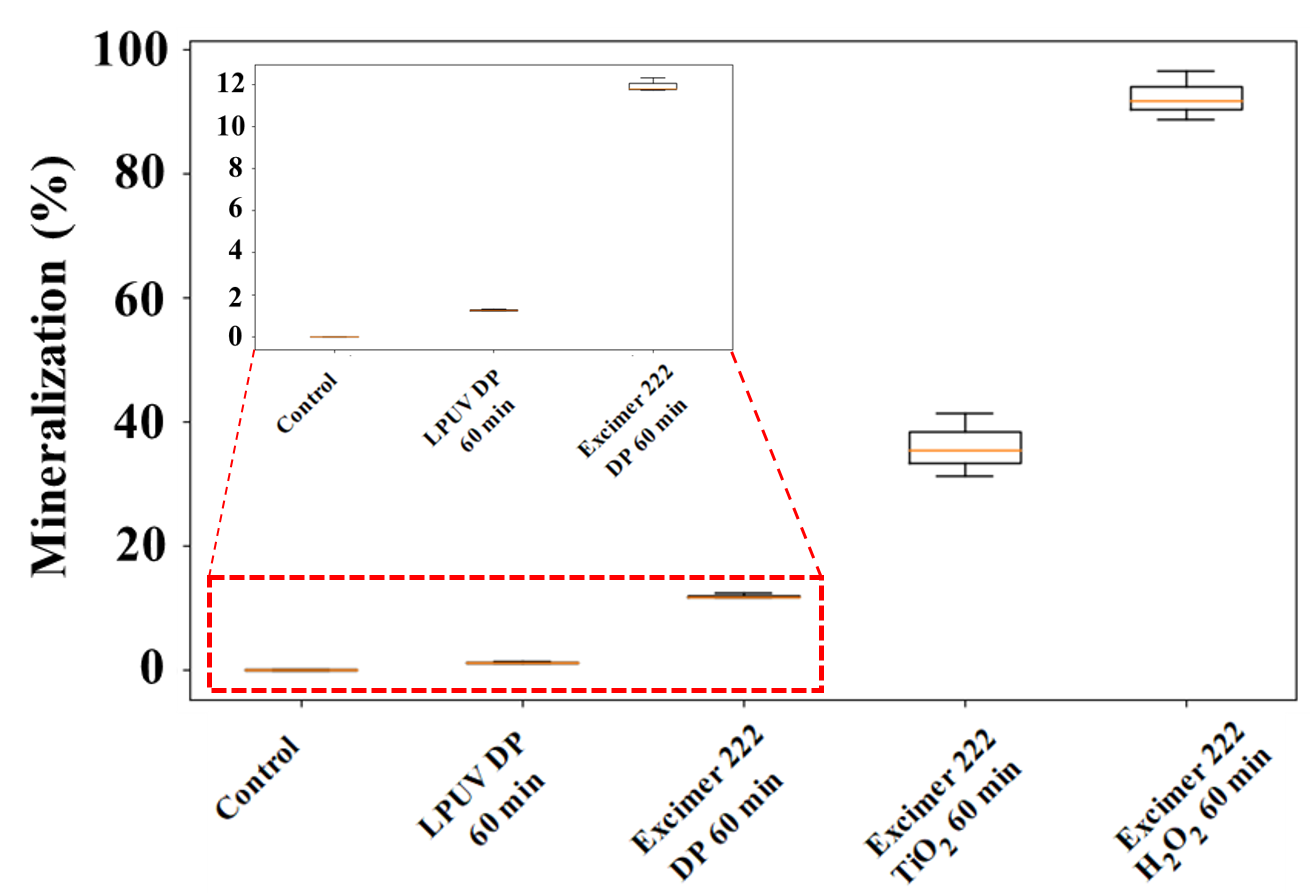


**Figure S4**: Mineralization as a function of different treatment methods ($C_{0}$ = 50 mg/L, 1g/L TiO_2_, and 10 ppm H_2_O_2_ and pH 10).


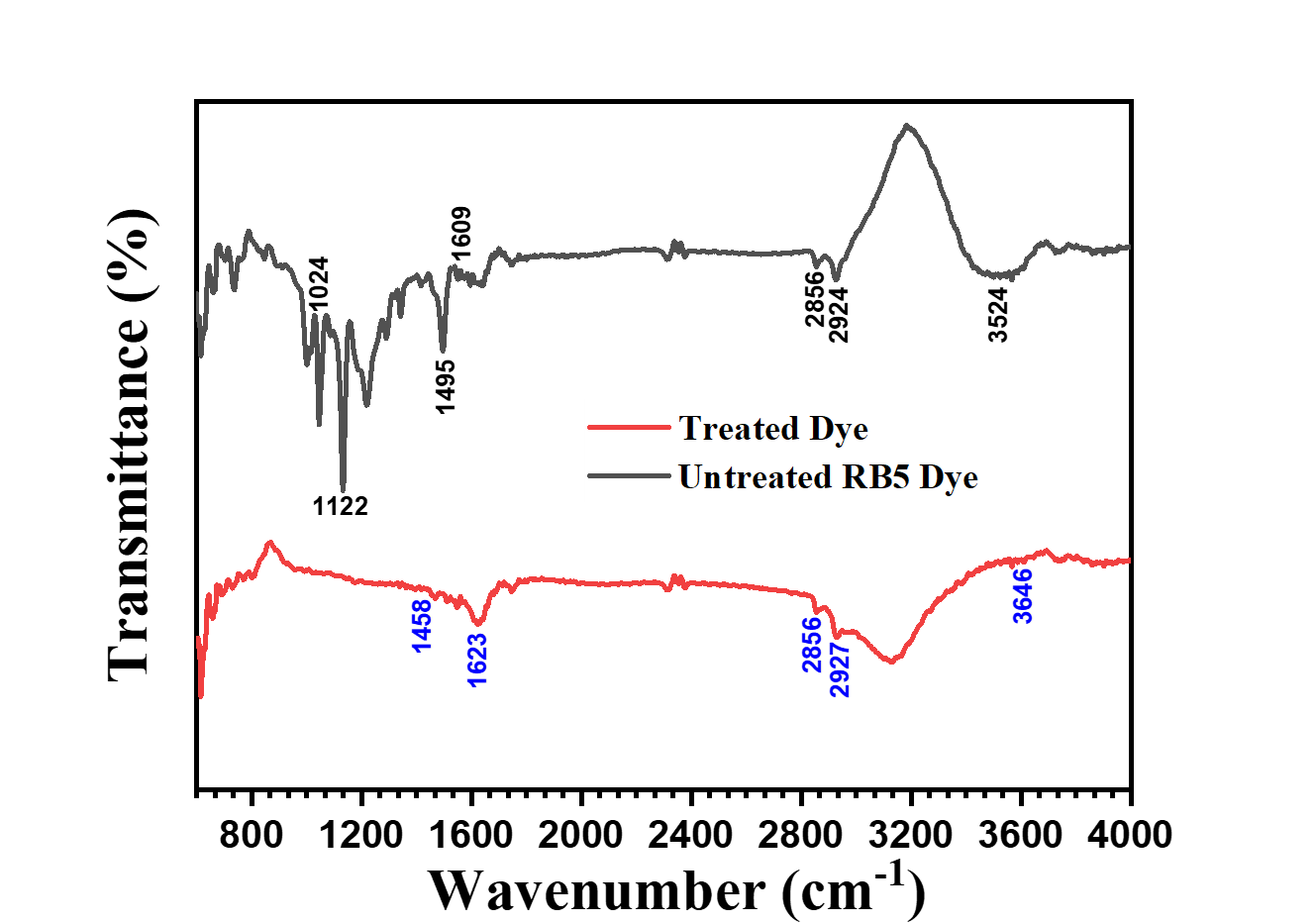


**Figure S5:** FTIR analysis of untreated and treated artificial wastewater (excimer 222/H_2_O_2_) samples.

**References**

(1) Ahlawat, K.; Jangra, R.; Ish, A.; Jain, N.; Prakash, R. A Dielectric Barrier Discharge Based Low Pressure Narrow Band Far UV-C 222 Nm Excimer Lamp and Its Efficiency Analysis. *Phys. Scr.* **2024**, *99* (2), 025018.
